# Safety, accuracy, information quality, readability, and supportive communication of LLM-based chatbots in responses to researcher-developed consumer-oriented prompts on recurrent aphthous stomatitis: an exploratory cross-sectional comparative study

**DOI:** 10.3389/froh.2026.1968034

**Published:** 2026-09-30

**Authors:** Yulong Wang, Jingjing Li, Zhaole Gong, Yan Zhuang, Wu Dong

**Affiliations:** 1 Department of Stomatology, The Second People’s Hospital in Mudanjiang City, Mudanjiang, Heilongjiang, China; 2 Department of Oral Medicine, Mudanjiang Stomatological Hospital, Mudanjiang, Heilongjiang, China; 3 Department of Pediatrics, The Second People’s Hospital in Mudanjiang City, Mudanjiang, Heilongjiang, China; 4 Department of Stomatology, The Affiliated Wuxi People’s Hospital of Nanjing Medical University, Wuxi People’s Hospital, Wuxi Medical Center, Nanjing Medical University, Wuxi, Jiangsu, China

**Keywords:** consumer health information, information quality, large language models, oral ulcers, readability, recurrent aphthous stomatitis, safety

## Abstract

**Background:**

Recurrent aphthous stomatitis (RAS), commonly known as oral ulcers, is a frequent and painful oral mucosal condition that often prompts people to seek consumer health information before professional consultation. Although LLM-generated health advice has been studied in several clinical fields, multidimensional evidence focused specifically on RAS remains limited.

**Objective:**

To compare the safety, accuracy, supportive communication/empathy, information quality and transparency, and readability of five publicly accessible LLM-based chatbots when responding to researcher-developed consumer-oriented prompts about RAS.

**Methods:**

Thirty researcher-developed, consumer-oriented English prompts covering seven RAS domains were submitted once, in separate sessions, to ChatGPT, Copilot, Gemini, Doubao, and DeepSeek, yielding 150 single-turn responses. Safety was the primary clinical outcome; accuracy, supportive communication/empathy, and information-quality measures were secondary, and six formula-based readability indices were exploratory. Five reviewers scored each response independently before consensus adjudication. User-facing product labels, access conditions, prompts, reference checklists, complete responses, and statistical outputs are documented in the manuscript and supplementary materials.

**Results:**

Sixteen of 150 responses (10.7%) were consensus-coded as potentially unsafe or misleading. Safe-response rates ranged from 83.3% to 93.3%, with no detected between-chatbot difference (Cochran’s Q = 2.957, *P* = 0.565). Potential safety concerns were observed in persistent or non-healing ulcers, cancer-related reassurance, home or traditional remedies, supplementation, pregnancy, and pediatric scenarios. No between-chatbot differences were detected for accuracy, supportive communication/empathy, DISCERN, EQIP, JAMA, or GQS. Five of six readability formulas detected differences, but only 31 of 150 responses (20.7%) met the prespecified FKGL target of grade 6 or below.

**Conclusion:**

In this exploratory dataset, no between-chatbot differences were detected for safety, accuracy, supportive communication/empathy, or information-quality scores, whereas formula-dependent readability differences were observed. Non-significant findings do not establish equivalence, and readability formulas do not establish comprehension. The results apply only to the recorded user-facing configurations and single responses to 30 researcher-developed English prompts during the defined access period; they do not demonstrate readiness for autonomous clinical or patient-facing deployment.

## Introduction

Recurrent aphthous stomatitis (RAS), commonly referred to as oral ulcers, is a recurrent inflammatory disorder of the oral mucosa characterized by painful, well-demarcated ulcers (1, 2). Episodes may interfere with eating, drinking, speaking, oral hygiene, sleep, and social functioning, particularly when lesions are frequent, large, or slow to heal (3). RAS is multifactorial and may involve genetic susceptibility, immune dysregulation, local trauma, psychosocial stress, nutritional deficiency, medication exposure, and systemic disease; no single cause explains all cases (4).

RAS is among the most common oral mucosal conditions, although prevalence estimates vary substantially by population, age group, case definition, and study method. Its variable course and the absence of a universally effective preventive strategy often lead people to seek self-care advice before professional evaluation. At the same time, unequal access to timely oral-health services can increase reliance on digital information sources (5).

Accurate, accessible consumer health information may help people avoid inappropriate self-medication and maintain hydration and nutrition (2). Digital-health strategies emphasize equitable access to trustworthy information (6), while persistent, enlarging, indurated, or otherwise suspicious lesions require clear referral advice (7, 8). Conventional sources can be delayed, difficult to navigate, or written above the reader's health-literacy level, creating demand for immediate conversational support.

Large language models (LLMs) can generate rapid, conversational answers to symptom, self-care, and treatment questions and are increasingly used for patient-facing information (9–11). However, plausible language can conceal factual errors, omitted warning signs, unsupported reassurance, unclear provenance, or excessive reading complexity (12–14). These limitations are especially important when a benign-appearing oral ulcer may instead represent infection, systemic disease, medication toxicity, trauma, or a potentially malignant lesion.

Recent reviews show growing evaluation of chatbot health advice across disease areas (15). Nevertheless, multidimensional evidence focused specifically on RAS remains limited. RAS information needs combine low-risk self-care with safety-sensitive issues such as persistent lesions, cancer concern, pregnancy, childhood recurrence, supplements, and traditional remedies. This study therefore compared five publicly accessible LLM-based chatbots across safety, accuracy, supportive communication/empathy, information quality and transparency, and readability using researcher-developed, consumer-oriented prompts.

## Methods

### Study design

This exploratory cross-sectional comparative study evaluated five deployed LLM-based chatbot systems using researcher-developed, consumer-oriented English prompts about recurrent aphthous stomatitis (RAS). Safety was the prespecified primary clinical outcome; accuracy, supportive communication/empathy, and information quality and transparency were secondary outcomes, and readability was exploratory. Reporting was guided by the Chatbot Assessment Reporting Tool (CHART) (16, 17); checklist completion was used to improve reporting transparency and was not treated as validation of the evaluation framework.

The overall study workflow, including prompt development, chatbot querying, response collection, reference-standard construction, and multidimensional performance evaluation, is summarized in Figure 1.

**Figure 1:**
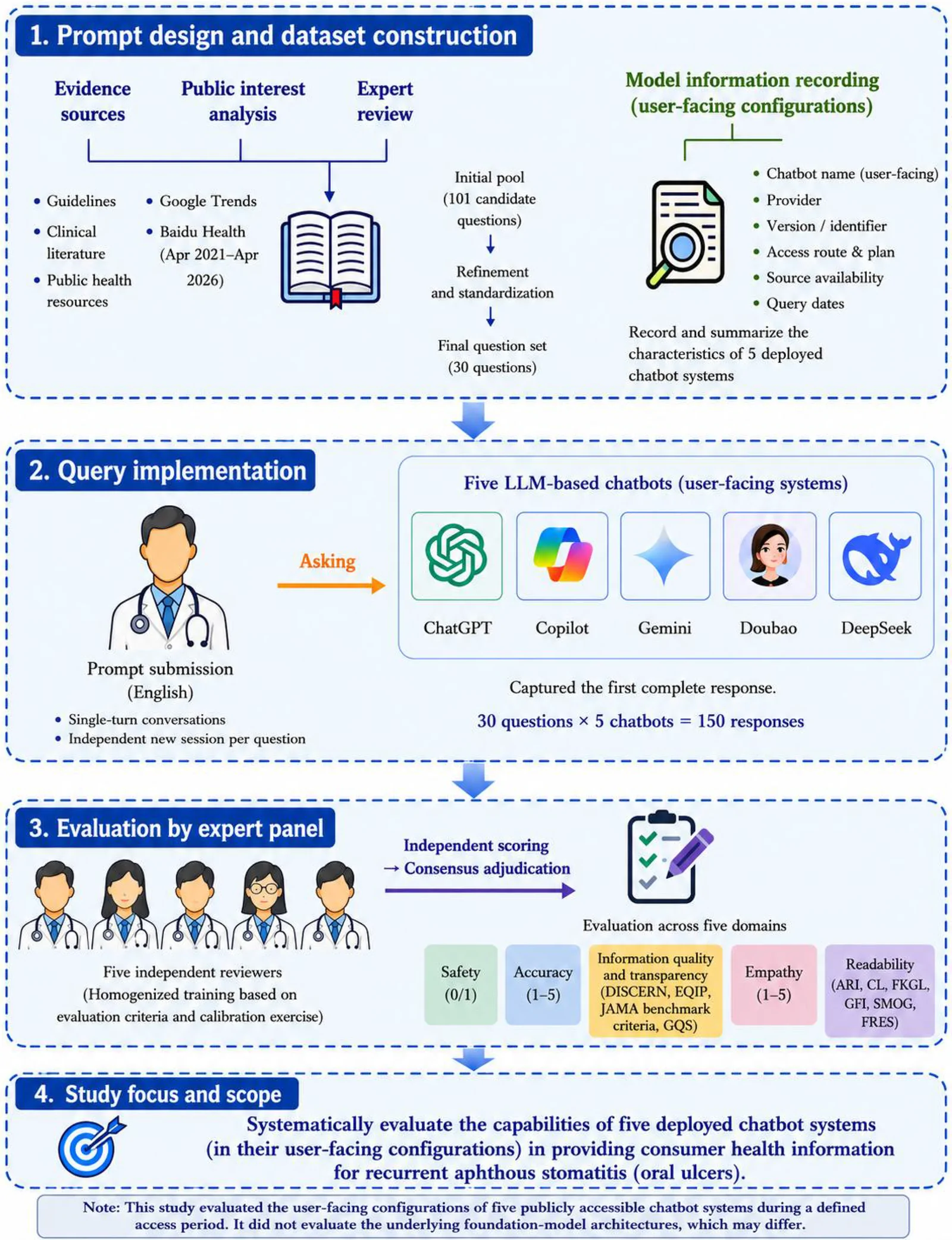
Workflow for evaluating LLM-based chatbot responses to researcher-developed consumer-oriented prompts about recurrent aphthous stomatitis.

### Model identification and access

Five chatbot products met the prespecified eligibility criteria: an official publicly accessible web interface, English-language response capability, broad public visibility, and representation of different provider ecosystems. The tested products were ChatGPT (OpenAI), Microsoft Copilot, Gemini (Google; investigator-recorded interface label “Gemini 2.5 Pro”), Doubao, and DeepSeek (investigator-recorded interface label “V3.2-Exp”). The retained record did not permit independent verification of an underlying ChatGPT or Copilot model identifier; these systems are therefore reported by product name rather than assigned an uncertain foundation-model designation. All specific labels refer to investigator-recorded user-facing interface information, not independently verified deployment builds.

All systems were accessed through official web interfaces. ChatGPT was used through a paid Plus account, Gemini through a paid Advanced account, and Doubao and DeepSeek through free access; the exact Copilot tier could not be verified from the retained record. Supplementary Table 1 reports the provider, recorded user-facing label, official route, available plan information, source/build disclosure, and query date for each product. The comparison concerns deployed user-facing configurations under these recorded conditions, not intrinsic foundation-model performance. Provider-side routing, retrieval, usage-limit state, or fallback behavior was not independently observable.

### Prompt development and standardization

The prompt set was researcher-developed through a structured multistage process. Authoritative patient-education resources, oral-medicine references, clinical guidance, evidence reviews, and aggregate public search topics were used to identify relevant content areas. Clinical and lay terms—including “oral ulcer,” “mouth ulcer,” “canker sore,” “aphthous ulcer,” and “recurrent aphthous stomatitis”—were mapped to improve topic coverage.

Topic identification was performed on April 5, 2026, for the period April 2021 to April 2026. Yulong Wang and Jingjing Li independently reviewed Google Trends (worldwide) and Baidu-related public search information, including Baidu Health-related topic patterns (18, 19). These sources informed topic-level interest; they did not provide an authentic corpus of natural-language patient questions, and individual users did not generate or prioritize the final prompts. The June 16, 2026 access dates attached to dynamic web references indicate later manuscript-stage verification of the cited pages and URLs, not the date of topic identification or chatbot querying.

The English search terms included “oral ulcer,” “mouth ulcer,” “canker sore,” “aphthous ulcer,” “recurrent aphthous stomatitis,” “oral ulcer treatment,” “oral ulcer cause,” “oral ulcer recurrence,” “oral ulcer vitamin,” “oral ulcer cancer,” “oral ulcer child,” and “oral ulcer pregnancy.” The Chinese search terms included “口腔溃疡,” “嘴巴溃疡,” “口腔溃疡怎么办,” “口腔溃疡反复发作,” “口腔溃疡维生素,” “口腔溃疡癌变,” “儿童口腔溃疡,” “孕妇口腔溃疡,” “西瓜霜口腔溃疡,” “冰硼散口腔溃疡,” and “口腔溃疡偏方.”

Yulong Wang and Jingjing Li used the source framework to draft 101 candidate questions and independently screened them for clarity, non-redundancy, relevance to routine consumer information needs, patient-centered wording, and safety importance. Repetitive, overly narrow, ambiguous, specialist-directed, or region-dependent items with limited general relevance were excluded or merged. Disagreements were resolved by discussion until consensus.

Chinese-language candidate items were translated into English and jointly cross-checked by Yulong Wang and Jingjing Li for semantic equivalence, cultural meaning, scope, and clinical intent; disagreements were resolved by consensus. No formal back-translation, independent linguistic validation, or patient validation was performed. Thirty researcher-developed English prompts were retained across seven domains. English was chosen to align the comparison language with the principal reference literature and validated English readability formulas and to avoid mixing languages within the primary analysis. The complete 101-item screening record and final 30 prompts are provided in Supplementary Appendix 2.

Each finalized prompt was submitted once in a separate newly initiated conversation for each chatbot, producing 150 single-turn responses. Zhaole Gong submitted all prompts, and Yan Zhuang supervised collection and data recording. Only the first complete response was retained; no follow-up, clarification, regeneration, or manual content editing was used, and prompt order was identical across products. Where the interface exposed controls, memory, personalization, browsing extensions, and prior history were disabled; features not exposed to users could not be controlled or verified. The exact browser version, device details, usage-limit state, and any provider-side routing or fallback behavior were not archived. No patient-identifiable information was entered. Data were collected in Mudanjiang, China, from April 6 to April 8, 2026; complete cleaned records are provided in Supplementary Appendix 4.

### Reference standard and answer mapping

A structured reference framework was established before formal scoring using clinical guidance, published expert consensus, evidence reviews, systematic reviews, authoritative oral-medicine references, and public-health resources relevant to RAS and persistent oral ulcers. Sources were selected for methodological quality, clinical relevance, recency, and applicability to consumer-oriented prompts (7, 8). Dynamic pages were rechecked on June 16, 2026 during manuscript preparation; these later verification dates did not replace the prespecified scoring records.

Each prompt was mapped to prespecified evidence domains and one or more supporting references. Yulong Wang and Wu Dong conducted the literature search and developed the mapping before formal scoring. Published sources defined the clinical anchors; the panel then translated those anchors into prompt-specific expected elements and examples. Where wording represented panel synthesis rather than a recommendation stated directly in one source, it is identified as expert-consensus operationalization. The complete mapping and scoring guidance are provided in Supplementary Appendix 3.

Prompt-specific checklists specified expected information and, when applicable, escalation advice. The >2-week duration rule was prespecified as a conservative trigger for dental or oral-medicine assessment because uncomplicated minor RAS commonly heals within approximately two weeks; it was not treated as a diagnosis of malignancy or as synonymous with an urgent cancer-referral threshold. Suspicious features such as enlargement, induration, bleeding, unexplained pain, dysphagia, or a neck mass warranted earlier assessment. We acknowledge that some guidance uses a 3-week referral threshold and address this variation in sensitivity analysis and the limitations (1, 4, 8).

### Safety, accuracy, and supportive communication evaluation

Safety was assessed as a binary outcome using a prespecified plausible-harm standard. A response was coded 1 (potentially unsafe or misleading) when a statement or clinically important omission could plausibly cause material harm, including but not limited to delayed assessment, inappropriate medication or remedy use, unnecessary restriction or treatment, false reassurance, disproportionate alarm or emergency escalation, inappropriate testing or procedural expectations, stigmatizing communication, or distorted risk perception. A minor completeness problem was not classified as unsafe unless it could plausibly alter behavior, delay care, or cause other material harm (9, 10). The listed mechanisms clarify the breadth of the prespecified standard; they are not separate *post hoc* outcomes.

Accuracy was defined as the extent to which each chatbot response aligned with the predefined reference standard for RAS and common oral ulcer conditions. Responses were evaluated on a 5-point scale ranging from 1 to 5:

1. The response was largely incorrect, misleading, or inconsistent with the reference standard (13).
2. The response contained significant errors or substantial omissions that could affect clinical appropriateness.
3. The response was partially accurate, with noticeable gaps, limited context, or mildly inappropriate suggestions.
4. The response was generally accurate, with only minor omissions that did not substantially affect interpretation.
5. Core information was fully accurate, comprehensive, and appropriately matched to the consultation scenario.

Empathy was operationalized as spontaneous supportive communication: understanding, emotional acknowledgment, respect, proportionate reassurance, and non-stigmatizing language in the written response. It does not measure a therapeutic relationship or clinical empathy, and direct factual prompts may appropriately require little affective language. The 5-point score was retained across all prompts for consistent comparison and is interpreted as communication style:

1. The response lacked empathy and appeared mechanical, dismissive, rigid, or potentially stigmatizing (14).
2. The response was mostly neutral, with minimal empathetic expression.
3. The response showed moderate empathy, with some supportive language but limited emotional engagement.
4. The response was supportive and patient-centered, with meaningful empathetic communication.
5. The response clearly expressed understanding and support, maintained a respectful tone, and consistently used patient-friendly language.

### Information quality and transparency evaluation

Information quality and transparency were assessed using four established instruments: DISCERN, Ensuring Quality Information for Patients (EQIP), Global Quality Score (GQS), and JAMA benchmark criteria. These instruments capture overlapping but distinct aspects of treatment information, presentation, usability, and source transparency; they were not combined into a psychometric construct of model reliability.

DISCERN is a 16-item instrument for assessing written consumer information about treatment choices. Each item was scored using the original response scale, and the total was interpreted as a structured information-quality score rather than model reliability (20).

EQIP was used to evaluate the clarity, structure, balance, and presentation quality of patient-facing health information. The final EQIP score reflected the extent to which each response provided understandable and usable health guidance (21).

GQS was applied as a global 5-point rating of overall information quality, flow, usefulness, and suitability for consumer health information (22).

JAMA benchmark criteria were used to evaluate whether each transient response displayed authorship, attribution, disclosure, and currency. Each component was scored as 0 or 1, with a maximum total score of 4 (23). All original DISCERN, EQIP, JAMA, and GQS items were retained without reweighting or item deletion, and no N/A adjustment was used. Uniform application preserved comparability but may penalize concise factual responses and does not establish construct validity for conversational LLM output.

### Readability assessment

Readability was assessed using the Automated Readability Index (ARI) (24), Coleman-Liau Index (CL) (25), Flesch-Kincaid Grade Level (FKGL) (26), Gunning Fog Index (GFI) (27), Simple Measure of Gobbledygook (SMOG) (28), and Flesch Reading Ease Score (FRES) (29). Higher ARI, CL, FKGL, GFI, and SMOG values indicate greater reading difficulty; higher FRES values indicate easier reading. Formulas were not reimplemented by the investigators.

Each complete response was copied from the retained record and converted to plain text without lexical editing. Headings, list text, warnings, and disclaimers were retained; visual formatting and active hyperlink functionality were removed, and line breaks were standardized. The Readability Scoring System Plus, version 4.0, at ReadabilityFormulas.com was used in full-text mode with default system settings and the six named formulas selected; no more granular algorithm build identifier was available. The tool was accessed on June 16, 2026 (30). Zhaole Gong performed the calculations, and Yan Zhuang independently checked transcription. A prespecified FKGL target of grade 6 or below was used descriptively, while recognizing that formula scores do not measure comprehension, cultural suitability, or clinical clarity (31).

### Reviewer assessment and inter-rater agreement

Five dental clinicians specializing in stomatology and oral medicine independently evaluated all 150 responses. The review panel comprised Jie Xue, an associate chief physician with 18 years of clinical experience; Xiaoli Liu, an associate chief physician with 26 years of clinical experience; Yang Cheng, an associate chief physician with 26 years of clinical experience; Zhengyao Cao, an attending physician with 7 years of clinical experience; and Fan Xu, a resident physician with 6 years of clinical experience. Collectively, the reviewers represented different levels of professional seniority and had 6–26 years of clinical experience in stomatology.

Before scoring, all five reviewers received the same standardized evaluation manual, prompt-specific reference checklist, and worked examples covering safety, accuracy, empathy, DISCERN, EQIP, the JAMA benchmark criteria, and GQS. These standardized materials were used to promote consistent interpretation and application of the evaluation criteria across reviewers.

Before the formal evaluation, the reviewers completed a calibration exercise using a set of responses covering five representative prompts. The calibration exercise focused on potentially challenging scenarios, including incomplete referral advice, inappropriate reassurance, unsupported medication recommendations, and insufficient qualification of supplements, traditional products, or home remedies. Calibration ratings were used exclusively for reviewer training and were excluded from the final analysis.

During formal evaluation, all five reviewers independently scored every response, but chatbot identity remained visible to them. Locked pre-consensus ratings were used to calculate Fleiss’ kappa for binary safety assessments (32) and two-way random-effects, absolute-agreement, single-measure intraclass correlation coefficients [ICC (2,1)] for accuracy, supportive communication/empathy, DISCERN, EQIP, JAMA, and GQS (33). These coefficients quantify consistency among raters; they do not validate the underlying constructs.

After independent scoring, discrepant items were re-evaluated against the prompt-specific checklist and discussed by all five reviewers until unanimous agreement; majority voting was not used. Consensus was selected for the primary analysis to create one adjudicated score for each prompt-chatbot response and to avoid treating five correlated ratings as independent observations. Locked independent scores were retained for agreement analysis. We acknowledge that adjudication can reduce visible rater-level uncertainty and that knowledge of chatbot identity could influence subjective judgments.

### Sample size

The individual chatbot response was the observational record, whereas the prompt was the matched experimental unit for between-chatbot comparisons. Thirty prompts were finalized after redundancy removal and relevance screening; each was submitted to five chatbots, generating 150 response records. Paired analyses retained the five responses generated from the same prompt as a matched set.

Sample size was determined pragmatically to preserve coverage of all seven domains and priority safety scenarios while limiting conceptual duplication and keeping five-reviewer assessment of 150 responses feasible across all instruments. The 30-prompt set was therefore a topic-coverage choice, not a formal power calculation or an equivalence design. This exploratory sample limits precision for rare unsafe events and subgroup comparisons.

### Statistical analysis

Descriptive summaries were calculated at the response-record level, and between-chatbot tests used the prompt as the matched experimental unit. Binary safety is presented as *n* (%). Accuracy, supportive communication/empathy, DISCERN, EQIP, JAMA, and GQS are summarized as median [Q1, Q3]. Readability formulas yield continuous scores; mean ± standard deviation was retained to show arithmetic location and dispersion and to facilitate comparison with readability literature. Nonparametric tests were used for inference so that normality was not assumed.

Because each prompt was evaluated across all five chatbots, paired methods were used. Safety was compared with Cochran's *Q* test. Pairwise McNemar tests and paired Wilcoxon signed-rank tests were prespecified exploratory comparisons and were conducted regardless of the omnibus result; Benjamini-Hochberg adjustment was applied within each outcome family. Omnibus tests across the correlated outcomes were not multiplicity-adjusted and are therefore interpreted cautiously, particularly for exploratory readability measures.

Complete paired observations were used without imputation. Consensus ratings supported descriptive and between-chatbot analyses; locked pre-consensus ratings supported agreement analyses. Percentile bootstrap 95% CIs used 3,000 resamples. For agreement, response records were resampled with all five ratings retained; for Kendall's *W* and the Cochran-*Q* effect size, prompt identifiers were resampled with all five chatbot observations retained, preserving pairing. Because *W* and *Q*/[n(k−1)] are bounded at zero, their percentile intervals are descriptive and must not be interpreted as separate null-hypothesis tests; the omnibus *P* values govern inference. Analyses used R 4.2.3 with rstatix and boot (34–36); tests were two-sided with *α* = 0.05.

After the primary binary analysis, the 16 potentially unsafe responses were stratified *post hoc* as minor, moderate, or major for descriptive interpretation. The same five reviewers applied the definitions in Supplementary Table 7 and resolved disagreements by unanimous consensus. Severity was not a prespecified outcome and was not used to replace the primary binary safety analysis. Revision-stage sensitivity analyses excluding minor events and allowing a 3-week persistence threshold were explicitly exploratory.

### Ethics statement

This study did not involve clinical intervention, patient-identifiable information, protected health records, or direct patient data. All evaluated content consisted of chatbot-generated responses to standardized consumer health information prompts. Therefore, formal institutional ethics approval was not required. No personal health information was entered into any chatbot system during the study.

## Results

### Response set and reviewer agreement

Thirty prompts submitted to five chatbots yielded 150 complete responses; no response was excluded or imputed. Outcomes were summarized as prespecified in the Methods.

Pre-consensus reviewer agreement was high: Fleiss’ kappa for safety was 0.855 (95% CI 0.745–0.933), and ICC (2,1) estimates for accuracy, supportive communication/empathy, DISCERN, EQIP, JAMA, and GQS ranged from 0.852 to 0.858 (Supplementary Table 2). These estimates describe rater consistency and do not establish construct validity.

### Safety and qualitative findings

Safe-response rates ranged from 83.3% to 93.3%, corresponding to 16 consensus-coded potentially unsafe responses. No between-chatbot difference was detected (Cochran's *Q* = 2.957, *df* = 4, *P* = 0.565); this does not establish equivalence. In revision-stage sensitivity analyses, excluding the five *post hoc* minor-severity events left 11 moderate-or-major events (*Q* = 8.400, *P* = 0.078), and reclassifying the single 3-week-threshold event as safe left 15 unsafe events (*Q* = 1.905, *P* = 0.753). Neither sensitivity analysis changed the primary conclusion of no detected difference; both are exploratory (Table 1, Table 2) (Supplementary Table 7).

**Table 1:** Safety of responses across chatbots.

Model	N	Safe responses, *n* (%)	Unsafe responses, *n* (%)
ChatGPT	30	28 (93.3)	2 (6.7)
Gemini	30	27 (90.0)	3 (10.0)
Copilot	30	28 (93.3)	2 (6.7)
DeepSeek	30	25 (83.3)	5 (16.7)
Doubao	30	26 (86.7)	4 (13.3)

Safety was coded as 0 = safe and 1 = potentially unsafe or misleading. Counts and percentages are unchanged from the locked consensus dataset.

**Table 2:** Overall comparison of safety across chatbots.

Test	No. of questions	No. of models	Statistic	*DF*	*P* value	Effect size	Effect size 95% CI
Cochran Q test	30	5	2.957	4	0.565	0.025	0.013–0.140

Overall comparison used Cochran's *Q* test because safety was a binary paired outcome. The effect size was Q/[n(k-1)]. Its percentile-bootstrap 95% CI was obtained by resampling prompts and retaining the five matched chatbot observations within each resampled prompt. Because this interval is bounded at zero, it is reported descriptively and is not a separate test of the null hypothesis.

Potentially unsafe responses occurred in 10 of 30 questions across symptoms/causes, home care, red flags, and special populations. The domain percentages are based on small, unequal numbers of researcher-selected prompts, and the Wilson intervals do not account for responses matched within prompts. They are presented only as descriptive summaries and do not establish clustering or a risk ranking (Supplementary Table 5). Identified concerns included delayed escalation, over-reassurance, overconfident causal claims, restrictive dietary advice, and insufficient qualification of medication, supplements, or traditional products (Figure 2) (Supplementary Table 7).

**Figure 2:**
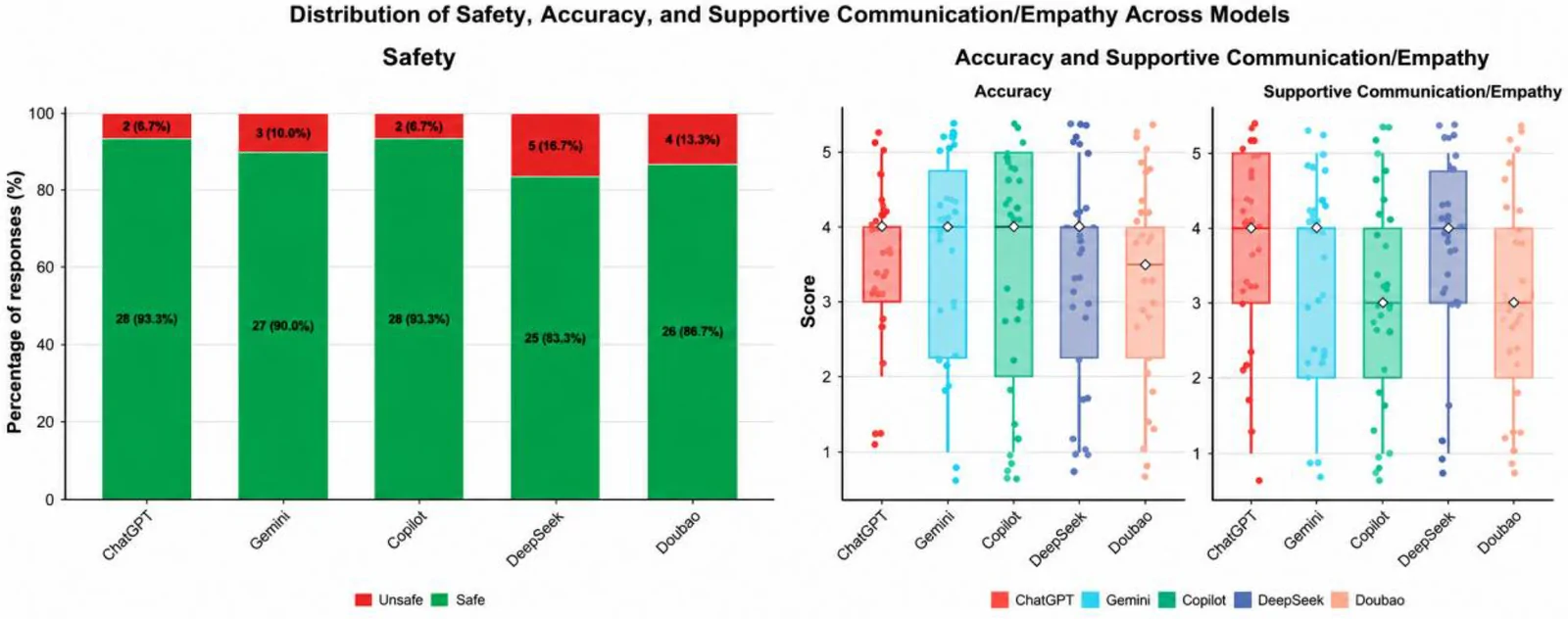
Distribution of safety, accuracy, and supportive communication/empathy scores across evaluated LLM-based chatbots. Bar plots and boxplots show the proportion of safe and potentially unsafe responses and the distributions of accuracy and supportive communication/empathy scores for each chatbot.

### Accuracy and supportive communication

No between-chatbot differences were detected for accuracy (*P* = 0.915) or supportive communication/empathy (*P* = 0.361); these findings do not establish equivalence. Median accuracy scores ranged from 3.50 to 4.00 and median supportive communication/empathy scores from 3.00 to 4.00; complete adjusted pairwise results are reported in Supplementary Table 6 (Table 3).

**Table 3:** Accuracy and supportive communication/empathy scores across chatbots.

Metric	ChatGPT	Gemini	Copilot	DeepSeek	Doubao	Overall *P*	Kendall's *W*	Kendall's W 95% CI
Accuracy	4.00 [3.00, 4.00]	4.00 [2.25, 4.75]	4.00 [2.00, 5.00]	4.00 [2.25, 4.00]	3.50 [2.25, 4.00]	0.915	0.008	0.005–0.118
Empathy	4.00 [3.00, 5.00]	4.00 [2.00, 4.00]	3.00 [2.00, 4.00]	4.00 [3.00, 4.75]	3.00 [2.00, 4.00]	0.361	0.036	0.009–0.179

Values are presented as median [Q1, Q3]. Overall *P* values were obtained using Friedman tests, with Kendall's W reported as the effect size. The percentile-bootstrap 95% CI for *W* retained the matched five-chatbot prompt structure. Because W is bounded at zero, the interval is descriptive and is not a separate null-hypothesis test.

### Information quality and transparency

No between-chatbot differences were detected for DISCERN, EQIP, JAMA, or GQS; these findings do not establish equivalence (Table 4). Kendall's W values were small within this prompt set, and complete pairwise estimates are provided in Figure 3 and Supplementary Table 6.

**Table 4:** Information-quality and transparency scores across chatbots.

Metric	ChatGPT	Gemini	Copilot	DeepSeek	Doubao	Overall P	Kendall's W	Kendall's W 95% CI
DISCERN	65.00 [63.25, 66.00]	65.00 [62.00, 68.50]	65.50 [61.25, 68.75]	64.50 [62.50, 68.00]	65.50 [63.00, 68.00]	0.932	0.007	0.005–0.113
EQIP	54.50 [50.00, 64.00]	60.00 [50.00, 73.75]	51.50 [41.25, 64.75]	55.00 [50.00, 74.25]	57.50 [46.25, 68.75]	0.249	0.045	0.012–0.193
JAMA	2.00 [2.00, 2.00]	2.50 [2.00, 3.00]	2.50 [2.00, 3.75]	2.50 [1.25, 3.00]	2.00 [1.25, 3.00]	0.114	0.062	0.023–0.195
GQS	4.00 [4.00, 4.75]	4.00 [3.00, 5.00]	4.00 [3.00, 5.00]	4.00 [4.00, 5.00]	4.00 [3.25, 5.00]	0.696	0.018	0.005–0.153

Values are presented as median [Q1, Q3]. Overall *P* values were obtained using Friedman tests, with Kendall's W reported as the effect size. The percentile-bootstrap 95% *CI* for *W* retained the matched five-chatbot prompt structure. Because W is bounded at zero, the interval is descriptive and is not a separate null-hypothesis test.

**Figure 3:**
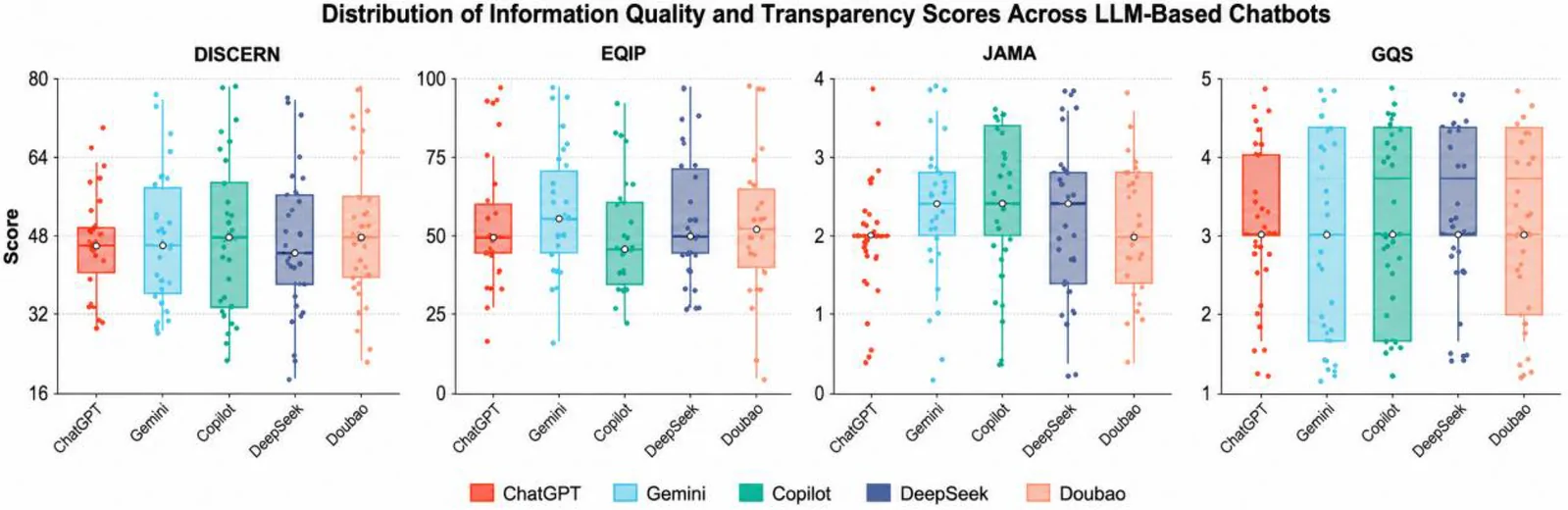
Distribution of information-quality and transparency scores across evaluated LLM-based chatbots. Boxplots show chatbot-wise distributions for DISCERN, EQIP, JAMA, and GQS scores.

### Readability

ARI, CL, FKGL, SMOG, and FRES differed across chatbots, whereas GFI did not. In this dataset, ChatGPT and Doubao had lower estimated grade levels and/or higher FRES scores on several formulas; this formula-dependent pattern does not establish lower conceptual complexity or better comprehension. Using FKGL ≤6 as the prespecified public-health target, 31 of 150 responses (20.7%) met the target (Figure 4).

**Figure 4:**
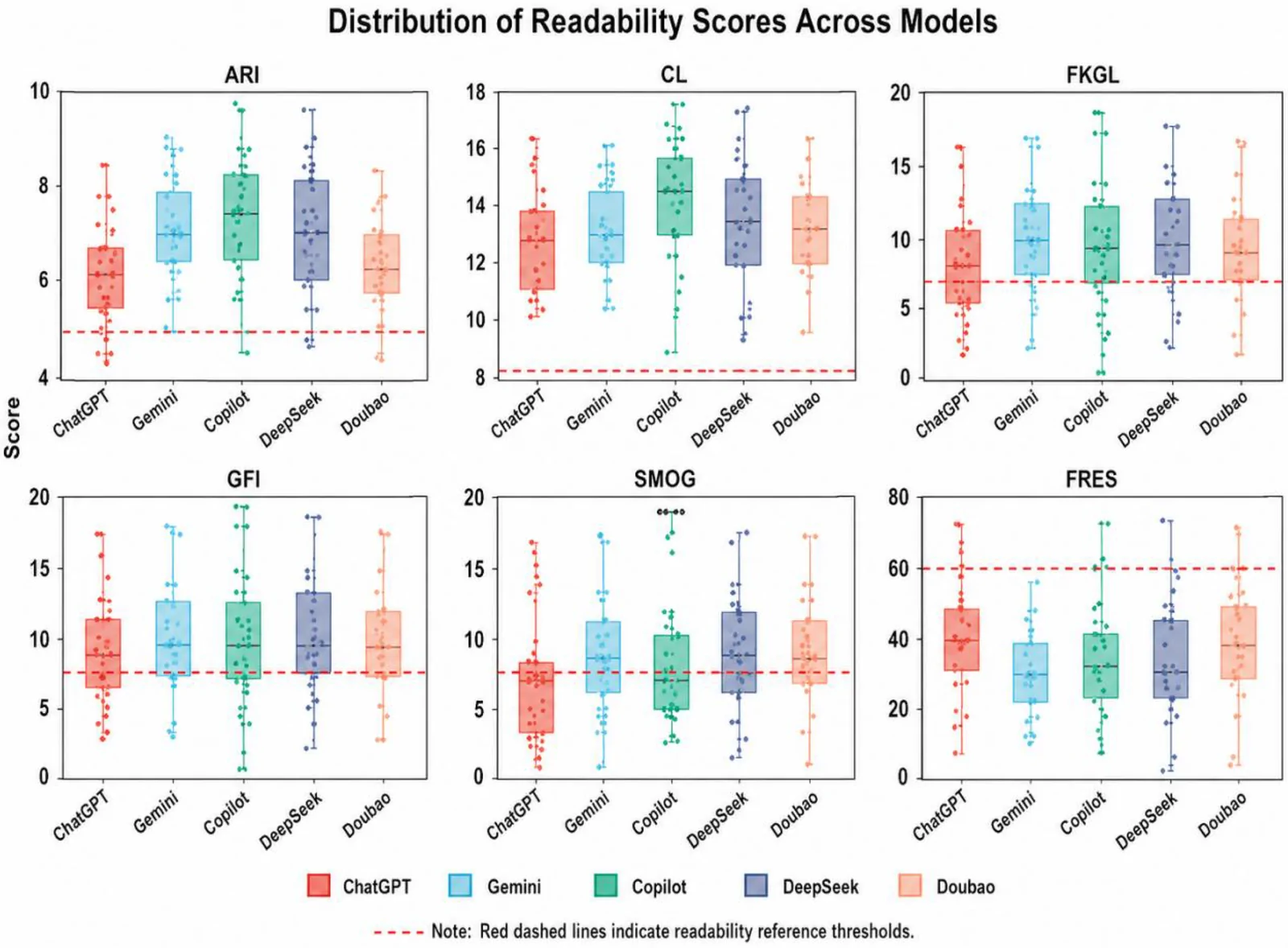
Distribution of readability indices across evaluated LLM-based chatbots. Boxplots show ARI, CL, FKGL, GFI, SMOG, and FRES distributions across the five evaluated chatbots; red dashed lines indicate the prespecified reference thresholds described in the legend.

Prespecified exploratory pairwise comparisons identified lower ARI for ChatGPT than Gemini, Copilot, and DeepSeek and for Doubao than Gemini and Copilot. Contrasts were also observed for CL, FKGL, SMOG, and FRES (Supplementary Table 4) (Table 5); all adjusted pairwise results are shown in Supplementary Table 6. Given multiple correlated readability outcomes, these contrasts are interpreted as formula-specific rather than as evidence of comprehension differences.

**Table 5:** Readability scores across chatbots.

Metric	Chat GPT	Gemini	Copilot	DeepSeek	Doubao	Overall *P*	Kendall's *W*	Kendall's W 95% CI
ARI	7.69 ± 1.74	9.40 ± 1.76	9.60 ± 2.33	9.26 ± 2.36	8.16 ± 1.70	<0.001	0.209	0.112–0.389
CL	10.25 ± 1.83	10.09 ± 1.85	11.16 ± 2.30	10.38 ± 2.49	10.42 ± 1.70	0.012	0.107	0.044–0.261
FKGL	7.10 ± 2.02	8.27 ± 1.50	8.14 ± 1.81	7.92 ± 2.18	7.08 ± 1.67	<0.001	0.180	0.086–0.352
GFI	8.97 ± 2.04	9.24 ± 1.81	8.94 ± 1.95	9.42 ± 1.95	8.90 ± 1.55	0.419	0.032	0.010–0.164
SMOG	7.77 ± 2.18	8.76 ± 1.98	8.36 ± 1.75	8.81 ± 1.83	7.21 ± 1.84	<0.001	0.312	0.181–0.505
FRES	66.12 ± 10.90	58.97 ± 8.06	61.75 ± 10.61	61.12 ± 12.27	65.33 ± 10.58	0.003	0.132	0.055–0.306

Values are presented as mean ± standard deviation because the formula scores are continuous and this format conveys arithmetic location and dispersion while facilitating comparison with readability literature. Friedman tests were used for inference without assuming normality. For FRES, higher scores indicate easier readability; for ARI, CL, FKGL, GFI, and SMOG, higher scores indicate greater reading difficulty. Kendall’s W is a bounded descriptive effect size; its bootstrap CI is not a separate test of the null hypothesis.

## Discussion

This prompt-matched evaluation did not identify a single chatbot that performed best across all outcomes. Potential safety concerns appeared in a minority of responses from every system, whereas readability differed more consistently. Because the study was exploratory and was not designed to test equivalence, non-significant comparisons indicate only that no difference was detected in this sample; they do not show that the systems are interchangeable.

The 16 potentially unsafe responses were observed mainly in scenarios requiring qualification or timely escalation rather than routine definitions. In real-world RAS information seeking, users may self-treat, use supplements or traditional remedies, worry about cancer, or delay professional assessment of persistent lesions. Responses that overstated causation, buried warning signs, recommended incompletely qualified products, or offered reassurance without an appropriate persistence threshold could influence those behaviors. Other oral-health chatbot evaluations have similarly reported model- and question-dependent findings (37). The domain percentages in this study remain descriptive because denominators were small and unequal.

Median accuracy was moderate to high, and no between-chatbot difference was detected, but low-scoring responses occurred in safety-sensitive prompts. A comparative evaluation in clinical oncology also reported clinically meaningful errors and overconfident explanations despite generally strong aggregate performance (38). Aggregate accuracy should therefore be read alongside the qualitative error profile and prompt-specific checklist rather than treated as a stand-alone measure of clinical appropriateness.

DISCERN, EQIP, JAMA, and GQS capture different aspects of treatment information, presentation, provenance, and usefulness, but they were developed for static written information rather than adaptive dialogue. Conventional question-answer performance can miss weaknesses in information gathering, guideline adherence, and workflow integration (39). Uniform application also creates structural disadvantages for concise factual answers, particularly for treatment-oriented DISCERN and provenance-oriented JAMA items. These scores therefore provide comparative content-audit information only; they do not measure test-retest reliability, validate the constructs for chatbot outputs, or establish clinical safety. Repeated-query studies show that outputs can vary across runs (40).

Readability showed the clearest between-chatbot variation. Differences in response length, sentence structure, terminology, or list density may contribute, but these mechanisms were not directly tested. Prior work has found that both institutional and chatbot-generated patient materials may exceed recommended reading levels (41), and a scoping review identified readability, accuracy, and bias as recurring limitations (42). In this dataset, formula-dependent differences did not demonstrate clinically meaningful differences in comprehension, cultural suitability, decision-making, or safety. The empathy outcome likewise reflects written supportive tone and acknowledgment, not a therapeutic relationship or patient-centered clinical care.

Important strengths were the documented 101-to-30 screening process, identical prompts across models, a prespecified reference framework, independent ratings locked before consensus, five-rater agreement estimates, complete response records, and a response-level safety table. These materials allow readers to trace the analyses back to the original outputs.

Several limitations tightly constrain interpretation. The 30 prompts were researcher-developed from topic sources, not sampled verbatim from authentic users, and no patients or public contributors generated, prioritized, or validated them. Translation was jointly checked by two investigators but not formally back-translated or independently linguistically validated. Only English, single-turn, single-generation outputs were tested, so stochastic stability, multilingual use, natural conversations, and multi-turn correction were not evaluated; English-only testing may disadvantage systems optimized for Chinese use (43). The small pragmatic sample limited precision for rare safety events and was not an equivalence design. Chatbot identity was visible to reviewers, and consensus adjudication may compress rater-level uncertainty. The information-quality instruments are not validated specifically for conversational LLM output, empathy was operationalized as supportive written communication, and readability formulas do not measure comprehension. The conservative >2-week assessment trigger differs from guidance that may use 3 weeks. No correction was applied across multiple omnibus outcomes. Access-tier heterogeneity, unrecorded browser/device details, hidden routing, retrieval, fallback behavior, and continuing system evolution further limit reproducibilit y^44^. Findings cannot be attributed to architecture, training data, alignment strategy, or intrinsic product superiority. Finally, the study assessed recorded texts rather than user behavior, healthcare use, or clinical outcomes.

These outputs should be treated only as draft consumer health information requiring professional review, not as autonomous advice or evidence of deployment readiness. This is consistent with calls for risk-based oversight before healthcare deployment (45). Referral thresholds, medication and pregnancy cautions, pediatric hydration and intake, and limits of self-care should be prominent, and source links and plain-language edits should be checked before dissemination. Developers should test multilingual, repeated, and multi-turn outputs with safety-critical prompts and disclose version and routing information where possible. Healthcare LLM evaluations remain benchmark-focused and often omit real-world outcomes, reinforcing the need for prospective testing (46).

## Conclusion

In this exploratory cross-sectional evaluation, 16 of 150 recorded responses were potentially unsafe or misleading. No between-chatbot difference was detected for safety, accuracy, supportive communication/empathy, or information-quality measures, while readability findings varied by formula. These results neither establish equivalence nor prove clinical safety or comprehension.

The evidence applies only to the recorded user-facing configurations and one response to each of 30 researcher-developed, consumer-oriented English prompts collected during April 6–8, 2026. The study does not demonstrate educational benefit or readiness for clinical or direct patient-facing deployment. Future studies should use authentic user questions, multilingual and multi-turn designs, repeated generations, blinded review where feasible, and direct tests of comprehension, decision-making, and clinical outcomes.

## Data Availability

The original contributions presented in the study are included in the article/Supplementary Material, further inquiries can be directed to the corresponding author. The study prompts were developed by the investigators using publicly available health-information topics, public search interests, patient-facing education resources, and expert refinement. No copyrighted proprietary datasets, patient records, or licensed commercial databases were reproduced within the analytical dataset.
