# Supplementary material for: Safety, accuracy, information quality, readability, and supportive communication of LLM-based chatbots in responses to researcher-developed consumer-oriented prompts on recurrent aphthous stomatitis: an exploratory cross-sectional comparative study
Source: Front Oral Health. 2026 Sep 30;7:1968034. doi: 10.3389/froh.2026.1968034 (PMC13661317; doi:10.3389/froh.2026.1968034)
Supplement: Supplementary file 1 [file Datasheet1.pdf]

CHART Checklist

Safety, Accuracy, Information Quality, Readability, and Supportive Communication of LLM-Based Chatbots in Responses to Researcher-Developed Consumer-Oriented Prompts on Recurrent Aphthous Stomatitis: An Exploratory Cross-Sectional Comparative Study

Interpretive note. Completion of CHART supports transparent reporting but does not validate the clinical or psychometric properties of the evaluation framework.

Heading	No.	CHART checklist item	Reporting location (section/table)
**Title and abstract**	1a	State that the study is assessing one or more generative AI-driven chatbots for clinical evidence or health advice.	Title
	1b	Apply a structured format, if applicable.	Structured Abstract
**Introduction**	2a	State the scientific background, rationale, and healthcare context for evaluating the generative AI-driven chatbot(s), referencing relevant literature when applicable.	Introduction
	2b	State the aims and research questions including the target audience, intervention, comparator(s), and outcome(s).	Abstract Objective; Introduction, final paragraph
**Methods**	3a	State the name and version identifier(s) of the generative AI model(s) and chatbot(s) under evaluation, as well as their date of release or last update.	Methods: Model Identification and Access; Supplementary Table S1 (user-facing product labels; unverified underlying model identifiers reported as unavailable)
	3b	State whether the generative AI model(s) and chatbot(s) are open-source or closed-source/proprietary.	Methods: Model Identification and Access; Supplementary Table S1
	4a	State whether the generative AI model was a base model or a novel base model, tuned model, or fine-tuned model.	Methods: Model Identification and Access; deployed user-facing configurations; tuning, routing, and build details unavailable when not disclosed
	4b	If a base model is used, cite its development in sufficient detail to identify the model.	Methods: Model Identification and Access; Supplementary Table S1
	4c	If a novel base model, tuned model, or fine-tuned model is used, describe the pre- and/or post-implementation/deployment data and parameters.	Not applicable to investigator development; unavailable proprietary deployment details are stated in Methods
**Prompt engineering**	5a	Describe the evolution of study prompt development.	Methods: Prompt Development and Standardization; Figure 1
	5ai	Describe the sources of prompts.	Methods: Prompt Development and Standardization; Supplementary Appendix 2
	5aii	State the number and characteristics of the individual(s) involved in prompt engineering.	Yulong Wang and Jingjing Li; Methods: Prompt Development and Standardization
	5aiii	Provide details of any patient and public involvement during prompt engineering.	No direct patient/public involvement; aggregate public search topics informed researcher-developed prompts; Methods and Limitations
	5b	Provide study prompts.	Final 30 prompts: Supplementary Appendix 2
**Query strategy**	6a	State route of access to generative AI model.	Methods: Model Identification and Access; Supplementary Table S1
	6b	State the date(s) and location(s) of queries for the generative AI-driven chatbot(s), including the day, month, and year as well as city and country.	Methods: Prompt Development and Standardization (Mudanjiang, China; April 6-8, 2026); dynamic source URLs were reverified June 16, 2026
	6c	Describe whether prompts were input into separate chat session(s).	Methods: Prompt Development and Standardization (separate session for each prompt-model pair)
	6d	Provide all generative AI-driven chatbot output/responses.	Complete cleaned responses: Supplementary Appendix 4
**Performance evaluation**	7a	Define the ground truth or reference standard used to define successful generative AI-driven chatbot performance.	Methods: Reference Standard and Answer Mapping; Supplementary Appendix 3
	7b	Describe the process undertaken for generative AI-driven chatbot performance evaluation.	Methods: Safety, Accuracy, and Supportive Communication; Information Quality and Transparency; Reviewer Assessment and Inter-Rater Agreement
	7bi	State the number and characteristics of team members involved in performance evaluation.	Methods: Reviewer Assessment and Inter-Rater Agreement (five-reviewer panel composition)
	7bii	Provide details of any patients and public involvement during the evaluation process.	No patient/public involvement in scoring; Methods and Ethics Statement
	7biii	State whether evaluators were blinded to the identity of the generative AI-driven chatbot(s) under assessment.	Methods: chatbot identity was visible during formal scoring; potential expectancy effects are acknowledged in Limitations

Heading	No.	CHART checklist item	Reporting location (section/table)
**Sample size**	8	Report how the sample size was determined.	Methods: Sample Size (30 prompts selected pragmatically; prompt is the matched experimental unit; no formal power/equivalence design)
**Data analysis**	9a	Describe statistical analysis methods, including any evaluation of reproducibility of generative AI-driven chatbot responses.	Methods: Statistical Analysis; single-generation design did not assess test-retest reproducibility and is explicitly limited
	9ai	Report the measures used for performance evaluation.	Methods: all performance measures; Supplementary Tables S2-S7
**Results**	10a	Report the performance evaluation undertaken including the alignment between generative AI-driven chatbot output and ground truth or reference standard using quantitative or mixed methods approaches as applicable.	Results; Tables 1-5; Figures 2-4; Supplementary Tables S2-S6
	10b	For responses deviating from the ground truth or reference standard, state the nature of the difference(s).	Results: Safety and qualitative findings; Supplementary Table S7
	10c	Report the evaluation for potentially harmful, biased, or misleading responses.	Results: Safety and qualitative findings; Supplementary Tables S5 and S7
**Discussion**	11a	Interpret study findings in the context of relevant evidence.	Discussion
	11b	Describe the strengths and limitations of the study.	Discussion: strengths and limitations
	11c	Describe the potential implications for practice, education, policy, regulation, and research.	Discussion: practice and research implications; Conclusion
**Open science**	12a	Report any relevant conflicts of interest for all authors.	Conflicts of interest
	12b	Report sources of funding and their role in the conduct and reporting of the study.	Funding
	12c	Describe the process undertaken for ethical approval.	Ethics Statement
	12ci	Describe the measures taken to safeguard data privacy of patient health information, as applicable.	Ethics Statement; Data privacy and confidentiality
	12cii	State whether permission/licensing was obtained for the use of original, copyrighted data.	Use of copyrighted materials
	12d	Provide a study protocol.	Protocol registration
	12e	State where study data, code repository, and model parameters can be accessed.	Data availability; Supplementary Appendices 1-4, Tables S1-S7, and data(5).xlsx. Proprietary parameters were unavailable.

Footer Note: AI, artificial intelligence; LLM, large language model; RAS, recurrent aphthous stomatitis.
