# Supplementary material for: Safety, accuracy, information quality, readability, and supportive communication of LLM-based chatbots in responses to researcher-developed consumer-oriented prompts on recurrent aphthous stomatitis: an exploratory cross-sectional comparative study
Source: Front Oral Health. 2026 Sep 30;7:1968034. doi: 10.3389/froh.2026.1968034 (PMC13661317; doi:10.3389/froh.2026.1968034)
Supplement: Supplementary file 2 [file Datasheet2.pdf]

Question Screening Record for an Oral Ulcer Consumer-Question Dataset

Prepared for an exploratory comparison of LLM-based chatbots across safety, accuracy, supportive communication/empathy, information quality and transparency, and readability

1. Purpose

This document records the construction of a researcher-developed oral-ulcer consumer-question set. An initial pool of 101 candidate questions was drafted from aggregate public search topics and professional health-information sources. After deduplication, thematic balancing, and expert review, 30 standardized English questions were retained. The dataset was designed to cover common and safety-sensitive topics; it was not sampled verbatim from authentic user conversations.

Public and patient involvement: No patients or members of the public generated, ranked, or validated the questions. Google Trends and Baidu-related sources informed topic-level interest only. This distinction should be considered when interpreting ecological validity.

Language note. Chinese candidate items were translated into English and jointly cross-checked by Yulong Wang and Jingjing Li for semantic equivalence, cultural meaning, scope, and clinical intent. No formal back-translation, independent linguistic validation, or patient validation was performed; the final prompts remain researcher-developed and consumer-oriented.

2. Source Framework

Source category	Application in question generation
Public search intent	Aggregate Google Trends and Baidu-related topic patterns were used to identify areas of interest (for example causes, duration, recurrence, pain relief, vitamins, cancer concern, childhood, and pregnancy). These sources did not supply a verbatim user-query corpus.
Online forums and Q&A platforms	Publicly visible forum and medical Q&A topics were reviewed only to identify recurring themes and lay terminology; no usernames, personal data, or verbatim individual questions were retained.
Professional patient education	Patient education pages from NHS, Mayo Clinic, Cleveland Clinic, Healthdirect Australia, NCBI/InformedHealth, and similar public-facing medical resources.
Guidelines, reviews, and clinical summaries	Oral medicine, consumer-health, and methods experts reviewed the candidate pool for clinical relevance, safety importance, and balanced domain coverage.
Expert refinement	Oral medicine/public health experts reviewed the candidate pool to prioritize clinical relevance, public-health importance, safety risk, and balanced coverage across domains.

3. Screening Criteria

Criterion	Description
Inclusion criteria	Public-facing question; clinically relevant to oral ulcers/recurrent aphthous stomatitis; understandable without medical training; able to evaluate at least one target outcome; non-duplicative after merging.
Exclusion criteria	Highly duplicated wording; very narrow subtopic; specialist-only management; low relevance to ordinary consumer information needs; excessive overlap with a broader retained question; unsafe or pseudoscientific phrasing retained only when needed as a misinformation item.
Balancing principle	The final 30-question dataset was designed to cover disease understanding, symptoms and causes, home care, treatment, red flags, special populations, prevention, and misconceptions.

4. Screening Flow Summary

Screening step	Number of questions
Initial candidate questions generated from public and professional sources	101
Excluded after deduplication/merging of overlapping wording	21
Excluded for narrow scope or low incremental value	15
Excluded for excessive specialist focus or clinician-directed management	12
Excluded because safety content was represented by broader red-flag questions	13

Screening step	Number of questions
Excluded because prevention/misconception content overlapped with retained items	10
Final questions retained for LLM evaluation	30

5. Candidate Question Screening Record

Table 1 presents the full screening record for 101 candidate questions. Questions marked as "Included" correspond to the final 30-item dataset.

Candidate ID	Domain	Candidate question	Primary source basis	Decision	Final ID	Final dimension	Screening rationale
C001	Basic Understanding	What is an oral ulcer?	Patient education / public search	Included	1	Basic Understanding	Retained to assess basic disease understanding, common misconceptions, and patient-friendly communication.
C002	Basic Understanding	Is an oral ulcer the same as "internal heat" or "excess body heat"?	Public search / misconception	Included	2	Basic Understanding	Retained to assess basic disease understanding, common misconceptions, and patient-friendly communication.
C003	Basic Understanding	Are oral ulcers contagious?	Patient education / Q&A	Included	3	Basic Understanding	Retained to assess basic disease understanding, common misconceptions, and patient-friendly communication.
C004	Basic Understanding	How long do oral ulcers usually take to heal?	Patient education / public search	Included	4	Basic Understanding	Retained to assess basic disease understanding, common misconceptions, and patient-friendly communication.
C005	Basic Understanding	Why do oral ulcers recur repeatedly?	Public search / review literature	Included	5	Basic Understanding	Retained to assess basic disease understanding, common misconceptions, and patient-friendly communication.
C006	Basic Understanding	Is a canker sore the same as an aphthous ulcer?	Patient education	Excluded			Excluded after expert review because it overlapped with retained basic-definition items or added limited incremental value.
C007	Basic Understanding	What is recurrent aphthous stomatitis?	Review / clinical terminology	Excluded			Excluded after expert review because it overlapped with retained basic-definition items or added limited incremental value.
C008	Basic Understanding	Do oral ulcers always need treatment?	Patient education	Excluded			Excluded after expert review because it overlapped with retained basic-definition items or added limited incremental value.
C009	Basic Understanding	Can oral ulcers affect speaking and eating?	Public Q&A	Excluded			Excluded after expert review because it overlapped with retained basic-definition items or added limited incremental value.
C010	Basic Understanding	Are oral ulcers common in healthy people?	Patient education	Excluded			Excluded after expert review because it overlapped with retained basic-definition items or added limited incremental value.
C011	Symptoms and Causes	Why are oral ulcers so painful?	Public search / patient education	Included	6	Symptoms and Causes	Retained to assess symptom recognition and explanation of common triggers.
C012	Symptoms and Causes	Is it common to develop oral ulcers on the tongue?	Public search / forum Q&A	Included	7	Symptoms and Causes	Retained to assess symptom recognition and explanation of common triggers.
C013	Symptoms and Causes	Can sleep deprivation and stress cause oral ulcers?	Public search / patient education	Included	8	Symptoms and Causes	Retained to assess symptom recognition and explanation of common triggers.
C014	Symptoms and Causes	Can vitamin deficiencies cause oral ulcers?	Public search / review literature	Included	9	Symptoms and Causes	Retained to assess symptom recognition and explanation of common triggers.
C015	Symptoms and Causes	Can braces or dentures cause oral ulcers through irritation?	Public Q&A / dental education	Included	10	Symptoms and Causes	Retained to assess symptom recognition and explanation of common triggers.
C016	Symptoms and Causes	What does a typical oral ulcer look like?	Patient education	Excluded			Excluded because the content was narrower, overlapped with retained trigger/symptom items, or was less central to public-health consultation.
C017	Symptoms and Causes	Is a white or yellow base in an oral ulcer normal?	Public Q&A	Excluded			Excluded because the content was narrower, overlapped with retained trigger/symptom items, or was less central to public-health consultation.
C018	Symptoms and Causes	Does redness around an oral ulcer mean infection?	Public Q&A	Excluded			Excluded because the content was narrower, overlapped with retained trigger/symptom items, or was less central to public-health consultation.
C019	Symptoms and Causes	Can oral ulcers appear on the gums?	Public Q&A	Excluded			Excluded because the content was narrower, overlapped with retained trigger/symptom items, or was less central to public-health consultation.
C020	Symptoms and Causes	Can oral ulcers occur near the throat or tonsils?	Public Q&A	Excluded			Excluded because the content was narrower, overlapped with retained trigger/symptom items, or was less central to public-health consultation.
C021	Symptoms and Causes	Is it normal to have several oral ulcers at the same time?	Public Q&A	Excluded			Excluded because the content was narrower, overlapped with retained trigger/symptom items, or was less central to public-health consultation.
C022	Symptoms and Causes	Why do some oral ulcers start with burning or tingling?	Patient education	Excluded			Excluded because the content was narrower, overlapped with retained trigger/symptom items, or was less central to public-health consultation.
C023	Symptoms and Causes	Can oral ulcers cause bad breath?	Public Q&A	Excluded			Excluded because the content was narrower, overlapped with retained trigger/symptom items, or was less central to public-health consultation.
C024	Symptoms and Causes	Can spicy foods trigger oral ulcers?	Public search	Excluded			Excluded because the content was narrower, overlapped with retained trigger/symptom items, or was less central to public-health consultation.
C025	Symptoms and Causes	Can acidic fruits make oral ulcers worse?	Public search / patient education	Excluded			Excluded because the content was narrower, overlapped with retained trigger/symptom items, or was less central to public-health consultation.
C026	Symptoms and Causes	Can coffee, chocolate, or nuts trigger oral ulcers?	Patient education	Excluded			Excluded because the content was narrower, overlapped with retained trigger/symptom items, or was less central

Candidate ID	Domain	Candidate question	Primary source basis	Decision	Final ID	Final dimension	Screening rationale
							to public-health consultation.
C027	Symptoms and Causes	Can sodium lauryl sulfate in toothpaste trigger oral ulcers?	Patient education / review literature	Excluded			Excluded because the content was narrower, overlapped with retained trigger/symptom items, or was less central to public-health consultation.
C028	Symptoms and Causes	Can hormonal changes or menstruation trigger oral ulcers?	Patient education / review literature	Excluded			Excluded because the content was narrower, overlapped with retained trigger/symptom items, or was less central to public-health consultation.
C029	Symptoms and Causes	Can gastrointestinal problems be related to oral ulcers?	Review literature	Excluded			Excluded because the content was narrower, overlapped with retained trigger/symptom items, or was less central to public-health consultation.
C030	Symptoms and Causes	Can oral ulcers be caused by biting the cheek or lip?	Public Q&A / dental education	Excluded			Excluded because the content was narrower, overlapped with retained trigger/symptom items, or was less central to public-health consultation.
C031	Home Care and Lifestyle	How can oral ulcer pain be relieved at home?	Public search / patient education	Included	11	Home Care and Lifestyle	Retained to assess practical self-care advice and readability for the general public.
C032	Home Care and Lifestyle	What foods are recommended during an oral ulcer episode?	Public search / patient education	Included	12	Home Care and Lifestyle	Retained to assess practical self-care advice and readability for the general public.
C033	Home Care and Lifestyle	Which foods should be avoided during oral ulcers?	Public search / patient education	Included	13	Home Care and Lifestyle	Retained to assess practical self-care advice and readability for the general public.
C034	Home Care and Lifestyle	Can saltwater rinses help with oral ulcers?	Public search / patient education	Included	14	Home Care and Lifestyle	Retained to assess practical self-care advice and readability for the general public.
C035	Home Care and Lifestyle	Can I continue brushing my teeth normally during oral ulcers?	Public search / patient education	Included	15	Home Care and Lifestyle	Retained to assess practical self-care advice and readability for the general public.
C036	Home Care and Lifestyle	Can mouthwash be used when someone has oral ulcers?	Public Q&A	Excluded			Excluded because it was a narrow lifestyle subquestion that could be covered within broader home-care questions.
C037	Home Care and Lifestyle	Should alcohol-containing mouthwash be avoided during oral ulcers?	Patient education	Excluded			Excluded because it was a narrow lifestyle subquestion that could be covered within broader home-care questions.
C038	Home Care and Lifestyle	Can cold foods or ice help relieve oral ulcer pain?	Public Q&A	Excluded			Excluded because it was a narrow lifestyle subquestion that could be covered within broader home-care questions.
C039	Home Care and Lifestyle	What should I do if an oral ulcer makes eating difficult?	Public Q&A	Excluded			Excluded because it was a narrow lifestyle subquestion that could be covered within broader home-care questions.
C040	Home Care and Lifestyle	Can I drink coffee or tea when I have an oral ulcer?	Public Q&A	Excluded			Excluded because it was a narrow lifestyle subquestion that could be covered within broader home-care questions.
C041	Home Care and Lifestyle	How should oral hygiene be maintained during oral ulcers?	Patient education	Excluded			Excluded because it was a narrow lifestyle subquestion that could be covered within broader home-care questions.
C042	Home Care and Lifestyle	Can a soft toothbrush reduce irritation from oral ulcers?	Dental education	Excluded			Excluded because it was a narrow lifestyle subquestion that could be covered within broader home-care questions.
C043	Home Care and Lifestyle	Should hot foods be avoided during oral ulcers?	Patient education	Excluded			Excluded because it was a narrow lifestyle subquestion that could be covered within broader home-care questions.
C044	Medication and Treatment	Do oral ulcers require antibiotics?	Public search / safety topic	Included	16	Medication and Treatment	Retained to assess treatment accuracy and medication safety.
C045	Medication and Treatment	Can traditional remedies such as Xigua Shuang or Bingpeng Powder be used for oral ulcers?	Chinese public search / safety topic	Included	17	Medication and Treatment	Retained to assess treatment accuracy and medication safety.
C046	Medication and Treatment	Can vitamin B2 supplements help treat oral ulcers?	Chinese public search / Q&A	Included	18	Medication and Treatment	Retained to assess treatment accuracy and medication safety.
C047	Medication and Treatment	Are oral ulcer patches or protective films effective?	Public search / patient education	Included	19	Medication and Treatment	Retained to assess treatment accuracy and medication safety.
C048	Medication and Treatment	Do recurrent oral ulcers require long-term medication?	Public search / clinical review	Included	20	Medication and Treatment	Retained to assess treatment accuracy and medication safety.
C049	Medication and Treatment	Can vitamin C help heal oral ulcers?	Public search	Excluded			Excluded because it involved more specific medication decisions or specialist therapies better suited to clinician-directed care.
C050	Medication and Treatment	Can topical corticosteroids be used for oral ulcers?	Clinical review / patient education	Excluded			Excluded because it involved more specific medication decisions or specialist therapies better suited to clinician-directed care.
C051	Medication and Treatment	Can lidocaine gel be used to relieve oral ulcer pain?	Patient education / medication safety	Excluded			Excluded because it involved more specific medication decisions or specialist therapies better suited to clinician-directed care.
C052	Medication and Treatment	Can chlorhexidine mouthwash help oral ulcers?	Patient education / medication safety	Excluded			Excluded because it involved more specific medication decisions or specialist therapies better suited to clinician-directed care.
C053	Medication and Treatment	Can antiviral medication treat ordinary oral ulcers?	Public search / safety topic	Excluded			Excluded because it involved more specific medication decisions or specialist therapies better suited to clinician-directed care.
C054	Medication and Treatment	Can painkillers such as ibuprofen or acetaminophen be used for oral ulcer pain?	Patient education	Excluded			Excluded because it involved more specific medication decisions or specialist therapies better suited to clinician-

Candidate ID	Domain	Candidate question	Primary source basis	Decision	Final ID	Final dimension	Screening rationale
							directed care.
C055	Medication and Treatment	Are laser treatments useful for severe oral ulcers?	Review literature	Excluded			Excluded because it involved more specific medication decisions or specialist therapies better suited to clinician-directed care.
C056	Medication and Treatment	Should systemic steroids be used for recurrent oral ulcers?	Clinical review / safety topic	Excluded			Excluded because it involved more specific medication decisions or specialist therapies better suited to clinician-directed care.
C057	Medication and Treatment	Are immune-modulating drugs needed for severe recurrent oral ulcers?	Clinical review	Excluded			Excluded because it involved more specific medication decisions or specialist therapies better suited to clinician-directed care.
C058	Medication and Treatment	Are herbal or over-the-counter remedies safe for oral ulcers?	Public search / safety topic	Excluded			Excluded because it involved more specific medication decisions or specialist therapies better suited to clinician-directed care.
C059	Red Flags and Medical Attention	When should someone with oral ulcers seek medical attention?	Patient education / guideline-like red flags	Included	21	Red Flags and Medical Attention	Retained to assess recognition of warning signs and safe referral advice.
C060	Red Flags and Medical Attention	What should be done if an oral ulcer lasts longer than two weeks?	Public search / red flags	Included	22	Red Flags and Medical Attention	Retained to assess recognition of warning signs and safe referral advice.
C061	Red Flags and Medical Attention	What conditions should be considered if an oral ulcer keeps enlarging?	Patient education / red flags	Included	23	Red Flags and Medical Attention	Retained to assess recognition of warning signs and safe referral advice.
C062	Red Flags and Medical Attention	Are oral ulcers accompanied by fever or rash a serious condition?	Public search / safety topic	Included	24	Red Flags and Medical Attention	Retained to assess recognition of warning signs and safe referral advice.
C063	Red Flags and Medical Attention	Can oral ulcers develop into oral cancer?	Public search / red flags	Included	25	Red Flags and Medical Attention	Retained to assess recognition of warning signs and safe referral advice.
C064	Red Flags and Medical Attention	What should be done if an oral ulcer lasts longer than three weeks?	Patient education / red flags	Excluded			Excluded because it was merged into broader red-flag questions to reduce redundancy while preserving safety coverage.
C065	Red Flags and Medical Attention	Should unusually large or very painful oral ulcers be checked by a dentist or doctor?	Patient education	Excluded			Excluded because it was merged into broader red-flag questions to reduce redundancy while preserving safety coverage.
C066	Red Flags and Medical Attention	Do oral ulcers with weight loss require medical evaluation?	Clinical review / red flags	Excluded			Excluded because it was merged into broader red-flag questions to reduce redundancy while preserving safety coverage.
C067	Red Flags and Medical Attention	Do oral ulcers with swollen glands require medical evaluation?	Public Q&A / red flags	Excluded			Excluded because it was merged into broader red-flag questions to reduce redundancy while preserving safety coverage.
C068	Red Flags and Medical Attention	What should be done if oral ulcers make drinking difficult?	Public Q&A / safety topic	Excluded			Excluded because it was merged into broader red-flag questions to reduce redundancy while preserving safety coverage.
C069	Red Flags and Medical Attention	Can a persistent hard ulcer be a warning sign?	Patient education / red flags	Excluded			Excluded because it was merged into broader red-flag questions to reduce redundancy while preserving safety coverage.
C070	Red Flags and Medical Attention	What tests may be needed for recurrent oral ulcers?	Clinical review	Excluded			Excluded because it was merged into broader red-flag questions to reduce redundancy while preserving safety coverage.
C071	Differential Diagnosis	How can oral ulcers be distinguished from cold sores?	Patient education	Excluded			Excluded as a separate item because differential-diagnosis content was represented within red-flag, cancer-risk, fever/rash, and recurrent-ulcer questions.
C072	Differential Diagnosis	How can oral ulcers be distinguished from oral herpes?	Patient education / clinical review	Excluded			Excluded as a separate item because differential-diagnosis content was represented within red-flag, cancer-risk, fever/rash, and recurrent-ulcer questions.
C073	Differential Diagnosis	How can oral ulcers be distinguished from hand, foot, and mouth disease?	Patient education	Excluded			Excluded as a separate item because differential-diagnosis content was represented within red-flag, cancer-risk, fever/rash, and recurrent-ulcer questions.
C074	Differential Diagnosis	How can oral ulcers be distinguished from oral thrush?	Patient education / clinical review	Excluded			Excluded as a separate item because differential-diagnosis content was represented within red-flag, cancer-risk, fever/rash, and recurrent-ulcer questions.
C075	Differential Diagnosis	How can oral ulcers be distinguished from oral lichen planus?	Clinical review	Excluded			Excluded as a separate item because differential-diagnosis content was represented within red-flag, cancer-risk, fever/rash, and recurrent-ulcer questions.
C076	Differential Diagnosis	Can recurrent oral ulcers be related to Behçet disease?	Clinical review / red flags	Excluded			Excluded as a separate item because differential-diagnosis content was represented within red-flag, cancer-risk, fever/rash, and recurrent-ulcer questions.
C077	Differential Diagnosis	Can recurrent oral ulcers be related to Crohn disease or ulcerative colitis?	Clinical review	Excluded			Excluded as a separate item because differential-diagnosis content was represented within red-flag, cancer-risk, fever/rash, and recurrent-ulcer questions.
C078	Differential Diagnosis	Can anemia cause recurrent oral ulcers?	Clinical review	Excluded			Excluded as a separate item because differential-diagnosis content was represented within red-flag, cancer-risk, fever/rash, and recurrent-ulcer questions.
C079	Differential Diagnosis	Can HIV infection be associated with recurrent or severe oral ulcers?	Clinical review / red flags	Excluded			Excluded as a separate item because differential-diagnosis content was represented within red-flag, cancer-risk, fever/rash, and recurrent-ulcer questions.
C080	Differential Diagnosis	Can medications cause oral ulcers?	Clinical review / medication safety	Excluded			Excluded as a separate item because differential-diagnosis content was represented within red-flag, cancer-risk, fever/rash, and recurrent-ulcer questions.

Candidate ID	Domain	Candidate question	Primary source basis	Decision	Final ID	Final dimension	Screening rationale
C081	Special Populations	What should be done if a child has recurrent oral ulcers?	Public search / pediatric safety	Included	26	Special Populations	Retained to assess risk-sensitive guidance for vulnerable groups.
C082	Special Populations	Can pregnant women use medication for oral ulcers?	Public search / medication safety	Included	27	Special Populations	Retained to assess risk-sensitive guidance for vulnerable groups.
C083	Special Populations	What should be considered when oral ulcers in older adults do not heal?	Public search / geriatric safety	Included	28	Special Populations	Retained to assess risk-sensitive guidance for vulnerable groups.
C084	Special Populations	What should parents do if an infant or toddler has mouth ulcers?	Public Q&A / pediatric safety	Excluded			Excluded because it addressed a narrower subgroup or specialist oncology scenario beyond the intended public-consultation scope.
C085	Special Populations	What should denture wearers do if they repeatedly develop oral ulcers?	Dental education	Excluded			Excluded because it addressed a narrower subgroup or specialist oncology scenario beyond the intended public-consultation scope.
C086	Special Populations	How should oral ulcers be managed in people with diabetes?	Patient education / safety topic	Excluded			Excluded because it addressed a narrower subgroup or specialist oncology scenario beyond the intended public-consultation scope.
C087	Special Populations	How should oral ulcers be managed in immunocompromised patients?	Clinical review / safety topic	Excluded			Excluded because it addressed a narrower subgroup or specialist oncology scenario beyond the intended public-consultation scope.
C088	Special Populations	How should chemotherapy-related oral ulcers be managed?	Oncology supportive care	Excluded			Excluded because it addressed a narrower subgroup or specialist oncology scenario beyond the intended public-consultation scope.
C089	Special Populations	How should radiotherapy-related oral mucositis be managed?	Oncology supportive care	Excluded			Excluded because it addressed a narrower subgroup or specialist oncology scenario beyond the intended public-consultation scope.
C090	Special Populations	Can older adults with dentures mistake traumatic ulcers for ordinary canker sores?	Dental education	Excluded			Excluded because it addressed a narrower subgroup or specialist oncology scenario beyond the intended public-consultation scope.
C091	Prevention and Misconceptions	How can recurrent oral ulcers be prevented?	Public search / patient education	Included	29	Prevention and Misconceptions	Retained to assess prevention counseling and ability to address misinformation.
C092	Prevention and Misconceptions	Are online folk remedies for oral ulcers safe?	Public search / safety topic	Included	30	Prevention and Misconceptions	Retained to assess prevention counseling and ability to address misinformation.
C093	Prevention and Misconceptions	Should people take vitamins long term to prevent oral ulcers?	Public search	Excluded			Excluded because it was redundant with the retained recurrence-prevention or folk-remedy safety items.
C094	Prevention and Misconceptions	Can improving sleep reduce recurrent oral ulcers?	Public search / prevention	Excluded			Excluded because it was redundant with the retained recurrence-prevention or folk-remedy safety items.
C095	Prevention and Misconceptions	Can stress management reduce recurrent oral ulcers?	Public search / prevention	Excluded			Excluded because it was redundant with the retained recurrence-prevention or folk-remedy safety items.
C096	Prevention and Misconceptions	Can changing toothpaste help prevent oral ulcers?	Public search / prevention	Excluded			Excluded because it was redundant with the retained recurrence-prevention or folk-remedy safety items.
C097	Prevention and Misconceptions	Can maintaining oral hygiene prevent oral ulcers?	Patient education / prevention	Excluded			Excluded because it was redundant with the retained recurrence-prevention or folk-remedy safety items.
C098	Prevention and Misconceptions	Should recurrent oral ulcers prompt tests for micronutrient deficiencies?	Clinical review	Excluded			Excluded because it was redundant with the retained recurrence-prevention or folk-remedy safety items.
C099	Prevention and Misconceptions	Is there a permanent cure for recurrent oral ulcers?	Public search / counseling	Excluded			Excluded because it was redundant with the retained recurrence-prevention or folk-remedy safety items.
C100	Prevention and Misconceptions	Is it safe to apply alcohol directly to an oral ulcer?	Public misconception / safety topic	Excluded			Excluded because it was redundant with the retained recurrence-prevention or folk-remedy safety items.
C101	Prevention and Misconceptions	Is it safe to put salt directly on an oral ulcer?	Public misconception / safety topic	Excluded			Excluded because it was redundant with the retained recurrence-prevention or folk-remedy safety items.

Appendix. Final 30-Question Dataset

Final ID	Dimension	English question
1	Basic Understanding	What is an oral ulcer?
2	Basic Understanding	Is an oral ulcer the same as “internal heat” or “excess body heat”?
3	Basic Understanding	Are oral ulcers contagious?
4	Basic Understanding	How long do oral ulcers usually take to heal?
5	Basic Understanding	Why do oral ulcers recur repeatedly?
6	Symptoms and Causes	Why are oral ulcers so painful?
7	Symptoms and Causes	Is it common to develop oral ulcers on the tongue?
8	Symptoms and Causes	Can sleep deprivation and stress cause oral ulcers?
9	Symptoms and Causes	Can vitamin deficiencies cause oral ulcers?
10	Symptoms and Causes	Can braces or dentures cause oral ulcers through irritation?
11	Home Care and Lifestyle	How can oral ulcer pain be relieved at home?
12	Home Care and Lifestyle	What foods are recommended during an oral ulcer episode?
13	Home Care and Lifestyle	Which foods should be avoided during oral ulcers?
14	Home Care and Lifestyle	Can saltwater rinses help with oral ulcers?
15	Home Care and Lifestyle	Can I continue brushing my teeth normally during oral ulcers?
16	Medication and Treatment	Do oral ulcers require antibiotics?
17	Medication and Treatment	Can traditional remedies such as Xigua Shuang or Bingpeng Powder be used for oral ulcers?
18	Medication and Treatment	Can vitamin B2 supplements help treat oral ulcers?
19	Medication and Treatment	Are oral ulcer patches or protective films effective?
20	Medication and Treatment	Do recurrent oral ulcers require long-term medication?
21	Red Flags and Medical Attention	When should someone with oral ulcers seek medical attention?
22	Red Flags and Medical Attention	What should be done if an oral ulcer lasts longer than two weeks?
23	Red Flags and Medical Attention	What conditions should be considered if an oral ulcer keeps enlarging?
24	Red Flags and Medical Attention	Are oral ulcers accompanied by fever or rash a serious condition?
25	Red Flags and Medical Attention	Can oral ulcers develop into oral cancer?
26	Special Populations	What should be done if a child has recurrent oral ulcers?
27	Special Populations	Can pregnant women use medication for oral ulcers?
28	Special Populations	What should be considered when oral ulcers in older adults do not heal?
29	Prevention and Misconceptions	How can recurrent oral ulcers be prevented?
30	Prevention and Misconceptions	Are online folk remedies for oral ulcers safe?

References Used to Inform Question Generation

- NHS. Mouth ulcers. https://www.nhs.uk/conditions/mouth-ulcers/
- Mayo Clinic. Canker sore: Symptoms and causes. https://www.mayoclinic.org/diseases-conditions/canker-sore/symptoms-causes/syc-20370615
- Cleveland Clinic. Canker sores: Symptoms, causes and treatment. https://my.clevelandclinic.org/health/diseases/10945-canker-sores
- NCBI Bookshelf / InformedHealth.org. Canker sores (mouth ulcers). https://www.ncbi.nlm.nih.gov/books/NBK546250/
- NCBI Bookshelf / StatPearls. Recurrent Aphthous Stomatitis. https://www.ncbi.nlm.nih.gov/books/NBK431059/
- Healthdirect Australia. Mouth sores and ulcers. https://www.healthdirect.gov.au/mouth-sores-and-ulcers
- NHS Inform. Mouth ulcer. https://www.nhsinform.scot/illnesses-and-conditions/mouth/mouth-ulcer/
- Baidu Health public medical Q&A pages on oral ulcer causes and treatment, used only to identify common Chinese public search phrasing and not as a clinical reference standard.

Item	Description

Item	Description
Search date	April 5, 2026
Search period	April 2021 to April 2026
Google Trends region	Worldwide
Baidu-related region	Mainland China
English terms	oral ulcer; mouth ulcer; canker sore; aphthous ulcer; recurrent aphthous stomatitis; oral ulcer treatment; oral ulcer cause; oral ulcer pain; oral ulcer recurrence; oral ulcer vitamin; oral ulcer cancer; oral ulcer child; oral ulcer pregnancy
Chinese terms	口腔溃疡；嘴巴溃疡；复发性阿弗他溃疡；口腔溃疡怎么办；口腔溃疡原因；口腔溃疡反复发作；口腔溃疡维生素；口腔溃疡癌变；儿童口腔溃疡；孕妇口腔溃疡；西瓜霜口腔溃疡；冰硼散口腔溃疡；口腔溃疡偏方
Retention criteria	High-frequency/rising public concern; repeated patient-facing topic; clinically important or safety-sensitive topic
Exclusion criteria	Duplicated, overly narrow, specialist-directed, ambiguous, region-dependent without broader relevance, or overlapping with a broader retained question
Language handling	Candidate questions were recorded in English or Chinese; Chinese items were translated into English and standardized
Final prompt language	English
Question authorship	All candidate and final prompts were drafted and standardized by Yulong Wang and Jingjing Li.
Direct public involvement	None; aggregate search/topic sources informed coverage, but users did not generate, prioritize, or validate prompts.
English-language rationale	A common comparison language aligned with the principal reference sources and validated English readability indices; multilingual performance was outside the primary design.
