# Supplementary material for: Safety, accuracy, information quality, readability, and supportive communication of LLM-based chatbots in responses to researcher-developed consumer-oriented prompts on recurrent aphthous stomatitis: an exploratory cross-sectional comparative study
Source: Front Oral Health. 2026 Sep 30;7:1968034. doi: 10.3389/froh.2026.1968034 (PMC13661317; doi:10.3389/froh.2026.1968034)
Supplement: Supplementary file 5 [file Datasheet5.pdf]

Supplementary Table S1. Characteristics of evaluated LLM-based chatbot systems

Configuration note. Labels identify the user-facing products/configurations recorded during access, not independently verified foundation-model architectures. ChatGPT Plus and Gemini Advanced were paid accounts; Doubao and DeepSeek used free access; the exact Copilot tier was not retained. Browser/device version, provider-side routing or retrieval, usage-limit state, and fallback behavior were not archived or independently observable. Each prompt was submitted once in a separate session, and user-controllable memory/personalization/browsing features were disabled where exposed.

Chatbot product	Provider	User-visible model identifier recorded at access	Official access route	Access plan	Source/build disclosure	Query dates
ChatGPT	OpenAI	ChatGPT (product label only; exact underlying model not displayed in the retained record)	Official ChatGPT web interface (Plus account)	Paid	Proprietary; weights, training data, and deployment build not publicly disclosed	2026-04-06
Copilot	Microsoft	Copilot (underlying model identifier not displayed)	Official web interface	Exact tier not retained or independently verifiable	Proprietary; underlying model and build not displayed	2026-04-06
Gemini	Google	Gemini 2.5 Pro (investigator-recorded user-facing label)	Official Gemini web interface (Advanced account)	Paid	Proprietary; deployment build not publicly disclosed	2026-04-06 to 2026-04-07
Doubao	Doubao AI, China	Doubao (underlying model identifier not displayed)	Official web interface	Free	Proprietary; underlying model and build not displayed	2026-04-07 to 2026-04-08
DeepSeek	DeepSeek AI, China	DeepSeek V3.2-Exp (investigator-recorded user-facing label; deployment build not further disclosed)	Official web interface	Free	Provider model label recorded; deployment build not further disclosed	2026-04-08

Supplementary Table S2. Inter-rater agreement

Metric	Agreement index	Estimate	95% CI	*P* value	Complete items	No. of raters	Note
Safety	Fleiss' kappa	0.855	0.745-0.933	<0.001	150	5	Primary agreement index for Safety; 95% CI based on 3000 nonparametric bootstrap resamples. A negative lower CI may occur when unsafe outcomes are extremely sparse because kappa can theoretically be below 0; this does not by itself indicate a coding error.
Accuracy	ICC(2,1)	0.852	0.817-0.883	<0.001	150	5	Two-way random-effects, absolute-agreement, single-measure ICC.
Empathy	ICC(2,1)	0.853	0.819-0.884	<0.001	150	5	Two-way random-effects, absolute-agreement, single-measure ICC.
DISCERN	ICC(2,1)	0.856	0.821-0.886	<0.001	150	5	Two-way random-effects, absolute-agreement, single-measure ICC.
EQIP	ICC(2,1)	0.858	0.825-0.888	<0.001	150	5	Two-way random-effects, absolute-agreement, single-measure ICC.
JAMA	ICC(2,1)	0.858	0.824-0.888	<0.001	150	5	Two-way random-effects, absolute-agreement, single-measure ICC.
GQS	ICC(2,1)	0.853	0.818-0.884	<0.001	150	5	Two-way random-effects, absolute-agreement, single-measure ICC.

Note: Safety was assessed using Fleiss' kappa. Accuracy, supportive communication/empathy, DISCERN, EQIP, JAMA, and GQS were assessed using two-way random-effects, absolute-agreement, single-measure ICC [ICC(2,1)]. Ninety-five percent confidence intervals used 3000 response-level percentile bootstrap resamples with all five ratings retained. Agreement quantifies rater consistency and does not establish construct validity.

Supplementary Table S3. Prespecified exploratory pairwise comparisons of safety

Comparison	N pairs	Safe rate difference, %	Safe rate difference 95% CI	Safe to unsafe, n	Unsafe to safe, n	Matched OR	Matched OR 95% *CI*	Adjusted *P*	Note
ChatGPT vs Gemini	30	3.33	-10.00-16.67	3	2	1.50	0.25-8.98	1.000	Pairwise McNemar test

Comparison	N pairs	Safe rate difference, %	Safe rate difference 95% CI	Safe to unsafe, n	Unsafe to safe, n	Matched OR	Matched OR 95% *CI*	Adjusted *P*	Note
ChatGPT vs Copilot	30	0.00	-10.00-10.00	1	1	1.00	0.06-15.99	1.000	Pairwise McNemar test
ChatGPT vs DeepSeek	30	10.00	-3.33-23.33	4	1	4.00	0.45-35.79	1.000	Pairwise McNemar test
ChatGPT vs Doubao	30	6.67	0.00-16.67	2	0	5.00	0.24-104.15	1.000	Pairwise McNemar test
Gemini vs Copilot	30	-3.33	-16.67-10.00	2	3	0.67	0.11-3.99	1.000	Pairwise McNemar test
Gemini vs DeepSeek	30	6.67	-10.00-23.33	4	2	2.00	0.37-10.92	1.000	Pairwise McNemar test
Gemini vs Doubao	30	3.33	-13.33-20.00	4	3	1.33	0.30-5.96	1.000	Pairwise McNemar test
Copilot vs DeepSeek	30	10.00	-3.33-26.67	4	1	4.00	0.45-35.79	1.000	Pairwise McNemar test
Copilot vs Doubao	30	6.67	0.00-16.67	2	0	5.00	0.24-104.15	1.000	Pairwise McNemar test
DeepSeek vs Doubao	30	-3.33	-20.00-13.33	3	4	0.75	0.17-3.35	1.000	Pairwise McNemar test

Note: Prespecified exploratory McNemar comparisons were conducted regardless of the omnibus-test result, with Benjamini-Hochberg correction within the safety outcome. Safe-rate differences and their bootstrap 95% CIs are reported. Existing numerical values are retained unchanged.

Supplementary Table S4. Significant prespecified exploratory pairwise comparisons for non-binary outcomes

Metric	Comparison	N pairs	Mean difference	Mean difference 95% CI	Median difference	Median difference 95% CI	Rank-biserial correlation	Rank-biserial 95% CI	Adjusted *P*
ARI	ChatGPT vs Gemini	30	-1.71	-2.31--1.11	-2.25	-2.56--1.05	-0.822	-0.983--0.585	0.001
ARI	ChatGPT vs Copilot	30	-1.91	-2.72--1.11	-1.86	-3.33--1.03	-0.746	-0.948--0.475	0.002
ARI	ChatGPT vs DeepSeek	30	-1.57	-2.31--0.88	-1.48	-2.23--0.26	-0.725	-0.931--0.419	0.002
ARI	Gemini vs Doubao	30	1.24	0.44-2.01	1.85	0.68-2.31	0.570	0.200-0.862	0.016
ARI	Copilot vs Doubao	30	1.43	0.32-2.45	2.21	0.95-2.73	0.507	0.140-0.837	0.031
CL	Gemini vs Copilot	30	-1.07	-1.73--0.32	-1.42	-2.04--0.82	-0.600	-0.914--0.239	0.042
FKGL	ChatGPT vs Gemini	30	-1.17	-1.85--0.38	-1.72	-2.33--0.74	-0.643	-0.948--0.282	0.019
FKGL	ChatGPT vs Copilot	30	-1.03	-1.69--0.31	-1.18	-2.03--0.40	-0.579	-0.871--0.213	0.019
FKGL	ChatGPT vs DeepSeek	30	-0.82	-1.57-0.04	-1.45	-2.02--0.43	-0.553	-0.880--0.161	0.019
FKGL	Gemini vs Doubao	30	1.20	0.50-1.86	1.62	0.58-2.21	0.598	0.265-0.888	0.019
FKGL	Copilot vs Doubao	30	1.06	0.37-1.80	1.12	0.20-2.02	0.544	0.187-0.850	0.019
SMOG	ChatGPT vs Gemini	30	-0.99	-1.84-0.01	-1.35	-1.77--0.74	-0.622	-0.961--0.234	0.008
SMOG	ChatGPT vs DeepSeek	30	-1.04	-1.97--0.05	-1.38	-2.13--0.46	-0.523	-0.850--0.140	0.026
SMOG	Gemini vs Doubao	30	1.55	0.53-2.57	1.72	0.90-2.32	0.654	0.290-0.979	0.006
SMOG	Copilot vs Doubao	30	1.15	0.67-1.61	1.00	0.64-1.80	0.802	0.523-0.987	<0.001

Metric	Comparison	N pairs	Mean difference	Mean difference 95% CI	Median difference	Median difference 95% CI	Rank-biserial correlation	Rank-biserial 95% CI	Adjusted *P*
SMOG	DeepSeek vs Doubao	30	1.60	0.97-2.25	1.64	0.49-2.08	0.841	0.587-0.987	<0.001
FRES	ChatGPT vs Gemini	30	7.14	3.54-11.35	5.00	0.98-9.00	0.717	0.397-0.936	0.012

Note: Prespecified exploratory paired Wilcoxon signed-rank comparisons were conducted regardless of the omnibus-test result, with Benjamini-Hochberg correction within each outcome. Paired mean/median differences and rank-biserial correlations are reported with bootstrap 95% CIs. Only adjusted P <0.05 comparisons are shown; numerical estimates are unchanged.

Supplementary Table S5. Domain-level distribution of consensus-coded potentially unsafe responses

Domain	Questions, n	Responses, n	Unsafe, n	Unsafe rate, %	Wilson 95% CI, %
Basic Understanding	5	25	0	0.0	0.0-13.3
Symptoms and Causes	5	25	4	16.0	6.4-34.7
Home Care and Lifestyle	5	25	6	24.0	11.5-43.4
Medication and Treatment	5	25	0	0.0	0.0-13.3
Red Flags and Medical Attention	5	25	3	12.0	4.2-30.0
Special Populations	3	15	3	20.0	7.0-45.2
Prevention and Misconceptions	2	10	0	0.0	0.0-27.8

Note: Data were derived from the locked consensus safety records in data(5).xlsx. The response is the observational record, and the prompt is the matched experimental unit for between-chatbot comparison. Potentially unsafe responses were identified using the final consensus safety code (0 = safe; 1 = potentially unsafe). Domain assignments followed Supplementary Appendix 2. Wilson 95% CIs are descriptive, are based on small and unequal domain denominators, and do not account for matching within prompts; they do not establish a risk ranking.

Supplementary Table S6. All prespecified exploratory pairwise comparisons for non-binary outcomes

Metric	Comparison	N pairs	Mean difference	Mean difference 95% CI	Median difference	Median difference 95% CI	Rank-biserial correlation	Rank-biserial 95% CI	Adjusted *P*	Note
Accuracy	ChatGPT vs Gemini	30	-0.10	-0.57-0.37	0.00	-1.00-0.00	-0.086	-0.553-0.391	0.982	Paired Wilcoxon signed-rank test
Accuracy	ChatGPT vs Copilot	30	0.13	-0.37-0.67	0.00	-1.00-1.00	0.087	-0.366-0.511	0.982	Paired Wilcoxon signed-rank test
Accuracy	ChatGPT vs DeepSeek	30	0.13	-0.40-0.70	0.00	-1.00-1.00	0.083	-0.362-0.536	0.982	Paired Wilcoxon signed-rank test
Accuracy	ChatGPT vs Doubao	30	0.17	-0.53-0.83	0.00	-1.00-1.00	0.135	-0.323-0.566	0.982	Paired Wilcoxon signed-rank test
Accuracy	Gemini vs Copilot	30	0.23	-0.30-0.77	0.00	-1.00-1.00	0.145	-0.304-0.540	0.982	Paired Wilcoxon signed-rank test
Accuracy	Gemini vs DeepSeek	30	0.23	-0.37-0.83	0.00	0.00-1.00	0.165	-0.333-0.632	0.982	Paired Wilcoxon signed-rank test
Accuracy	Gemini vs Doubao	30	0.27	-0.27-0.87	0.00	-1.00-1.00	0.187	-0.265-0.585	0.982	Paired Wilcoxon signed-rank test
Accuracy	Copilot vs DeepSeek	30	0.00	-0.60-0.60	0.00	0.00-0.00	-0.012	-0.556-0.515	0.982	Paired Wilcoxon signed-rank test

Metric	Comparison	N pairs	Mean difference	Mean difference 95% CI	Median difference	Median difference 95% CI	Rank-biserial correlation	Rank-biserial 95% CI	Adjusted *P*	Note
Accuracy	Copilot vs Doubao	30	0.03	-0.70-0.73	0.00	-1.00-1.00	0.021	-0.410-0.481	0.982	Paired Wilcoxon signed-rank test
Accuracy	DeepSeek vs Doubao	30	0.03	-0.63-0.70	0.00	-1.00-1.00	0.027	-0.424-0.475	0.982	Paired Wilcoxon signed-rank test
Empathy	ChatGPT vs Gemini	30	0.27	-0.30-0.87	0.00	-1.00-1.00	0.154	-0.277-0.575	0.629	Paired Wilcoxon signed-rank test
Empathy	ChatGPT vs Copilot	30	0.50	-0.13-1.10	0.50	0.00-1.00	0.307	-0.133-0.695	0.313	Paired Wilcoxon signed-rank test
Empathy	ChatGPT vs DeepSeek	30	-0.07	-0.57-0.53	0.00	-1.00-0.00	-0.094	-0.642-0.395	0.821	Paired Wilcoxon signed-rank test
Empathy	ChatGPT vs Doubao	30	0.63	0.00-1.33	0.00	-1.00-1.50	0.367	-0.082-0.720	0.313	Paired Wilcoxon signed-rank test
Empathy	Gemini vs Copilot	30	0.23	-0.40-0.83	0.00	0.00-1.00	0.169	-0.329-0.605	0.629	Paired Wilcoxon signed-rank test
Empathy	Gemini vs DeepSeek	30	-0.33	-0.87-0.23	0.00	-1.00-0.00	-0.312	-0.728-0.138	0.313	Paired Wilcoxon signed-rank test
Empathy	Gemini vs Doubao	30	0.37	-0.10-0.83	0.00	0.00-1.00	0.336	-0.105-0.725	0.313	Paired Wilcoxon signed-rank

**Metric**	**Comparison**	**N pairs**	**Mean difference**	**Mean difference 95% CI**	**Median difference**	**Median difference 95% CI**	**Rank-biserial correlation**	**Rank-biserial 95% CI**	**Adjusted *P***	**Note**
										test
Empathy	Copilot vs DeepSeek	30	-0.57	-1.30-0.13	0.00	-1.50-0.00	-0.351	-0.745-0.127	0.313	Paired Wilcoxon signed-rank test
Empathy	Copilot vs Doubao	30	0.13	-0.40-0.70	0.00	-1.00-1.00	0.047	-0.391-0.501	0.849	Paired Wilcoxon signed-rank test
Empathy	DeepSeek vs Doubao	30	0.70	-0.00-1.37	1.00	-0.50-2.00	0.398	0.005-0.738	0.313	Paired Wilcoxon signed-rank test
DISCERN	ChatGPT vs Gemini	30	-0.20	-2.23-1.70	-1.00	-3.00-2.00	-0.060	-0.466-0.343	0.983	Paired Wilcoxon signed-rank test
DISCERN	ChatGPT vs Copilot	30	-0.47	-2.40-1.47	-0.50	-3.00-3.00	-0.074	-0.485-0.363	0.983	Paired Wilcoxon signed-rank test
DISCERN	ChatGPT vs DeepSeek	30	-0.17	-2.40-1.90	-0.50	-3.00-2.00	-0.028	-0.428-0.396	0.983	Paired Wilcoxon signed-rank test
DISCERN	ChatGPT vs Doubao	30	-0.80	-3.00-1.30	-1.00	-3.00-2.50	-0.116	-0.505-0.292	0.983	Paired Wilcoxon signed-rank test
DISCERN	Gemini vs Copilot	30	-0.27	-2.33-1.67	0.50	-2.00-3.00	0.032	-0.390-0.460	0.983	Paired Wilcoxon signed-rank test

Metric	Comparison	N pairs	Mean difference	Mean difference 95% CI	Median difference	Median difference 95% CI	Rank-biserial correlation	Rank-biserial 95% CI	Adjusted *P*	Note
DISCERN	Gemini vs DeepSeek	30	0.03	-2.10-2.33	-1.00	-3.00-1.50	-0.039	-0.492-0.370	0.983	Paired Wilcoxon signed-rank test
DISCERN	Gemini vs Doubao	30	-0.60	-2.40-1.07	0.50	-2.00-1.00	-0.071	-0.458-0.348	0.983	Paired Wilcoxon signed-rank test
DISCERN	Copilot vs DeepSeek	30	0.30	-1.70-2.33	-0.50	-3.50-1.50	0.007	-0.432-0.421	0.983	Paired Wilcoxon signed-rank test
DISCERN	Copilot vs Doubao	30	-0.33	-1.93-1.20	-0.50	-2.00-1.50	-0.069	-0.483-0.350	0.983	Paired Wilcoxon signed-rank test
DISCERN	DeepSeek vs Doubao	30	-0.63	-2.60-1.23	-1.50	-3.00-2.50	-0.094	-0.492-0.344	0.983	Paired Wilcoxon signed-rank test
EQIP	ChatGPT vs Gemini	30	-3.43	-10.50-3.90	-3.00	-10.00-2.50	-0.208	-0.618-0.230	0.632	Paired Wilcoxon signed-rank test
EQIP	ChatGPT vs Copilot	30	3.63	-2.63-10.30	1.50	-2.50-7.50	0.199	-0.231-0.616	0.632	Paired Wilcoxon signed-rank test
EQIP	ChatGPT vs DeepSeek	30	-1.70	-8.40-5.03	-2.00	-10.00-5.00	-0.119	-0.547-0.320	0.666	Paired Wilcoxon signed-rank test
EQIP	ChatGPT vs Doubao	30	0.37	-7.93-9.03	0.00	-8.50-7.50	0.013	-0.411-0.440	0.962	Paired Wilcoxon signed-rank

Metric	Comparison	N pairs	Mean difference	Mean difference 95% CI	Median difference	Median difference 95% CI	Rank-biserial correlation	Rank-biserial 95% CI	Adjusted *P*	Note
										test
EQIP	Gemini vs Copilot	30	7.07	-0.10-14.53	5.00	0.00-15.00	0.381	-0.017-0.751	0.632	Paired Wilcoxon signed-rank test
EQIP	Gemini vs DeepSeek	30	1.73	-5.20-8.73	5.00	-5.00-9.00	0.143	-0.276-0.561	0.666	Paired Wilcoxon signed-rank test
EQIP	Gemini vs Doubao	30	3.80	-2.17-9.77	5.00	-2.00-10.00	0.276	-0.169-0.670	0.632	Paired Wilcoxon signed-rank test
EQIP	Copilot vs DeepSeek	30	-5.33	-11.40-0.77	-5.00	-10.00-1.00	-0.330	-0.705-0.087	0.632	Paired Wilcoxon signed-rank test
EQIP	Copilot vs Doubao	30	-3.27	-10.07-3.80	-5.00	-13.00-6.00	-0.217	-0.613-0.196	0.632	Paired Wilcoxon signed-rank test
EQIP	DeepSeek vs Doubao	30	2.07	-5.73-9.63	5.00	-9.50-12.00	0.116	-0.336-0.527	0.666	Paired Wilcoxon signed-rank test
JAMA	ChatGPT vs Gemini	30	-0.50	-0.93--0.07	-1.00	-1.00-0.00	-0.435	-0.825--0.012	0.205	Paired Wilcoxon signed-rank test
JAMA	ChatGPT vs Copilot	30	-0.53	-1.10--0.03	0.00	-1.00-0.00	-0.468	-0.837--0.014	0.205	Paired Wilcoxon signed-rank test

Metric	Comparison	N pairs	Mean difference	Mean difference 95% CI	Median difference	Median difference 95% CI	Rank-biserial correlation	Rank-biserial 95% CI	Adjusted *P*	Note
JAMA	ChatGPT vs DeepSeek	30	-0.43	-0.83--0.03	-0.50	-1.00-0.00	-0.462	-0.870--0.006	0.205	Paired Wilcoxon signed-rank test
JAMA	ChatGPT vs Doubao	30	-0.20	-0.60-0.17	0.00	-1.00-0.00	-0.212	-0.638-0.240	0.533	Paired Wilcoxon signed-rank test
JAMA	Gemini vs Copilot	30	-0.03	-0.63-0.57	0.00	-1.00-1.00	0.040	-0.391-0.487	0.867	Paired Wilcoxon signed-rank test
JAMA	Gemini vs DeepSeek	30	0.07	-0.40-0.53	0.00	0.00-1.00	0.100	-0.368-0.574	0.867	Paired Wilcoxon signed-rank test
JAMA	Gemini vs Doubao	30	0.30	-0.17-0.77	0.00	0.00-1.00	0.290	-0.190-0.698	0.476	Paired Wilcoxon signed-rank test
JAMA	Copilot vs DeepSeek	30	0.10	-0.40-0.60	0.00	-1.00-1.00	0.060	-0.405-0.490	0.867	Paired Wilcoxon signed-rank test
JAMA	Copilot vs Doubao	30	0.33	-0.20-0.87	1.00	0.00-1.00	0.268	-0.170-0.655	0.476	Paired Wilcoxon signed-rank test
JAMA	DeepSeek vs Doubao	30	0.23	-0.23-0.73	0.00	0.00-1.00	0.221	-0.228-0.652	0.533	Paired Wilcoxon signed-rank test
GQS	ChatGPT vs Gemini	30	0.03	-0.37-0.43	0.50	-1.00-1.00	0.014	-0.385-0.438	0.957	Paired Wilcoxon signed-rank

Metric	Comparison	N pairs	Mean difference	Mean difference 95% CI	Median difference	Median difference 95% CI	Rank-biserial correlation	Rank-biserial 95% CI	Adjusted *P*	Note
										test
GQS	ChatGPT vs Copilot	30	-0.10	-0.40-0.20	0.00	0.00-0.00	-0.181	-0.697-0.396	0.957	Paired Wilcoxon signed-rank test
GQS	ChatGPT vs DeepSeek	30	-0.13	-0.50-0.20	0.00	-1.00-0.00	-0.175	-0.647-0.307	0.957	Paired Wilcoxon signed-rank test
GQS	ChatGPT vs Doubao	30	-0.03	-0.47-0.40	0.00	-1.00-1.00	-0.051	-0.480-0.400	0.957	Paired Wilcoxon signed-rank test
GQS	Gemini vs Copilot	30	-0.13	-0.60-0.37	0.00	-1.00-0.00	-0.067	-0.563-0.397	0.957	Paired Wilcoxon signed-rank test
GQS	Gemini vs DeepSeek	30	-0.17	-0.63-0.30	0.00	-1.00-0.50	-0.147	-0.567-0.310	0.957	Paired Wilcoxon signed-rank test
GQS	Gemini vs Doubao	30	-0.07	-0.43-0.33	0.00	0.00-0.00	-0.081	-0.625-0.462	0.957	Paired Wilcoxon signed-rank test
GQS	Copilot vs DeepSeek	30	-0.03	-0.43-0.37	0.00	-1.00-0.50	-0.035	-0.505-0.433	0.957	Paired Wilcoxon signed-rank test
GQS	Copilot vs Doubao	30	0.07	-0.37-0.50	0.00	0.00-1.00	0.037	-0.453-0.543	0.957	Paired Wilcoxon signed-rank test

Metric	Comparison	N pairs	Mean difference	Mean difference 95% CI	Median difference	Median difference 95% CI	Rank-biserial correlation	Rank-biserial 95% CI	Adjusted *P*	Note
GQS	DeepSeek vs Doubao	30	0.10	-0.30-0.50	0.00	0.00-1.00	0.104	-0.364-0.550	0.957	Paired Wilcoxon signed-rank test
ARI	ChatGPT vs Gemini	30	-1.71	-2.31--1.11	-2.25	-2.56--1.05	-0.822	-0.983--0.585	0.001	Paired Wilcoxon signed-rank test
ARI	ChatGPT vs Copilot	30	-1.91	-2.72--1.11	-1.86	-3.33--1.03	-0.746	-0.948--0.475	0.002	Paired Wilcoxon signed-rank test
ARI	ChatGPT vs DeepSeek	30	-1.57	-2.31--0.88	-1.48	-2.23--0.26	-0.725	-0.931--0.419	0.002	Paired Wilcoxon signed-rank test
ARI	ChatGPT vs Doubao	30	-0.47	-1.33-0.29	-0.18	-1.02-0.44	-0.157	-0.557-0.273	0.574	Paired Wilcoxon signed-rank test
ARI	Gemini vs Copilot	30	-0.19	-0.88-0.50	-0.46	-1.05-0.10	-0.191	-0.614-0.241	0.536	Paired Wilcoxon signed-rank test
ARI	Gemini vs DeepSeek	30	0.14	-0.68-1.00	0.30	-0.96-1.19	0.058	-0.338-0.460	0.789	Paired Wilcoxon signed-rank test
ARI	Gemini vs Doubao	30	1.24	0.44-2.01	1.85	0.68-2.31	0.570	0.200-0.862	0.016	Paired Wilcoxon signed-rank test
ARI	Copilot vs DeepSeek	30	0.34	-0.57-1.24	0.64	-0.92-1.37	0.131	-0.273-0.523	0.597	Paired Wilcoxon signed-rank

Metric	Comparison	N pairs	Mean difference	Mean difference 95% CI	Median difference	Median difference 95% CI	Rank-biserial correlation	Rank-biserial 95% CI	Adjusted *P*	Note
										test
ARI	Copilot vs Doubao	30	1.43	0.32-2.45	2.21	0.95-2.73	0.507	0.140-0.837	0.031	Paired Wilcoxon signed-rank test
ARI	DeepSeek vs Doubao	30	1.10	0.14-2.09	0.95	-0.32-2.31	0.414	0.025-0.752	0.088	Paired Wilcoxon signed-rank test
CL	ChatGPT vs Gemini	30	0.16	-0.46-0.78	-0.07	-0.94-0.76	0.043	-0.368-0.445	0.845	Paired Wilcoxon signed-rank test
CL	ChatGPT vs Copilot	30	-0.91	-1.78-0.04	-1.43	-2.63--0.51	-0.503	-0.847--0.097	0.092	Paired Wilcoxon signed-rank test
CL	ChatGPT vs DeepSeek	30	-0.14	-0.83-0.57	-0.36	-1.23-1.09	-0.101	-0.497-0.303	0.845	Paired Wilcoxon signed-rank test
CL	ChatGPT vs Doubao	30	-0.18	-0.97-0.61	-0.34	-1.12-0.94	-0.067	-0.467-0.346	0.845	Paired Wilcoxon signed-rank test
CL	Gemini vs Copilot	30	-1.07	-1.73--0.32	-1.42	-2.04--0.82	-0.600	-0.914--0.239	0.042	Paired Wilcoxon signed-rank test
CL	Gemini vs DeepSeek	30	-0.29	-0.94-0.33	-0.14	-0.99-0.44	-0.140	-0.518-0.290	0.845	Paired Wilcoxon signed-rank test

Metric	Comparison	N pairs	Mean difference	Mean difference 95% CI	Median difference	Median difference 95% CI	Rank-biserial correlation	Rank-biserial 95% CI	Adjusted *P*	Note
CL	Gemini vs Doubao	30	-0.33	-1.10-0.37	-0.23	-1.27-0.71	-0.144	-0.557-0.260	0.845	Paired Wilcoxon signed-rank test
CL	Copilot vs DeepSeek	30	0.78	-0.16-1.65	0.79	0.16-1.87	0.424	0.028-0.763	0.146	Paired Wilcoxon signed-rank test
CL	Copilot vs Doubao	30	0.73	-0.32-1.68	1.30	-0.02-2.07	0.385	-0.011-0.750	0.168	Paired Wilcoxon signed-rank test
CL	DeepSeek vs Doubao	30	-0.04	-0.89-0.79	-0.10	-1.56-1.12	-0.050	-0.458-0.374	0.845	Paired Wilcoxon signed-rank test
FKGL	ChatGPT vs Gemini	30	-1.17	-1.85--0.38	-1.72	-2.33--0.74	-0.643	-0.948--0.282	0.019	Paired Wilcoxon signed-rank test
FKGL	ChatGPT vs Copilot	30	-1.03	-1.69--0.31	-1.18	-2.03--0.40	-0.579	-0.871--0.213	0.019	Paired Wilcoxon signed-rank test
FKGL	ChatGPT vs DeepSeek	30	-0.82	-1.57-0.04	-1.45	-2.02--0.43	-0.553	-0.880--0.161	0.019	Paired Wilcoxon signed-rank test
FKGL	ChatGPT vs Doubao	30	0.02	-0.83-0.94	-0.26	-0.67-0.71	-0.019	-0.424-0.387	0.934	Paired Wilcoxon signed-rank test
FKGL	Gemini vs Copilot	30	0.14	-0.44-0.69	0.34	-0.26-0.56	0.191	-0.226-0.591	0.457	Paired Wilcoxon signed-rank

Metric	Comparison	N pairs	Mean difference	Mean difference 95% CI	Median difference	Median difference 95% CI	Rank-biserial correlation	Rank-biserial 95% CI	Adjusted *P*	Note
										test
FKGL	Gemini vs DeepSeek	30	0.35	-0.30-1.03	0.66	-0.25-0.86	0.234	-0.191-0.613	0.381	Paired Wilcoxon signed-rank test
FKGL	Gemini vs Doubao	30	1.20	0.50-1.86	1.62	0.58-2.21	0.598	0.265-0.888	0.019	Paired Wilcoxon signed-rank test
FKGL	Copilot vs DeepSeek	30	0.21	-0.43-0.89	0.31	-0.18-0.59	0.149	-0.286-0.579	0.543	Paired Wilcoxon signed-rank test
FKGL	Copilot vs Doubao	30	1.06	0.37-1.80	1.12	0.20-2.02	0.544	0.187-0.850	0.019	Paired Wilcoxon signed-rank test
FKGL	DeepSeek vs Doubao	30	0.84	-0.00-1.72	1.10	-0.43-1.98	0.344	-0.080-0.682	0.170	Paired Wilcoxon signed-rank test
GFI	ChatGPT vs Gemini	30	-0.27	-1.06-0.66	-0.55	-1.28-0.12	-0.286	-0.671-0.114	0.709	Paired Wilcoxon signed-rank test
GFI	ChatGPT vs Copilot	30	0.03	-0.71-0.81	-0.35	-0.95-0.48	-0.101	-0.510-0.301	0.802	Paired Wilcoxon signed-rank test
GFI	ChatGPT vs DeepSeek	30	-0.45	-1.28-0.43	-0.85	-1.75-0.68	-0.224	-0.602-0.196	0.709	Paired Wilcoxon signed-rank test

Metric	Comparison	N pairs	Mean difference	Mean difference 95% CI	Median difference	Median difference 95% CI	Rank-biserial correlation	Rank-biserial 95% CI	Adjusted *P*	Note
GFI	ChatGPT vs Doubao	30	0.07	-0.77-0.87	0.25	-0.95-1.18	0.030	-0.350-0.439	0.894	Paired Wilcoxon signed-rank test
GFI	Gemini vs Copilot	30	0.30	-0.40-1.03	0.22	-0.60-0.66	0.154	-0.282-0.547	0.792	Paired Wilcoxon signed-rank test
GFI	Gemini vs DeepSeek	30	-0.18	-0.98-0.62	-0.10	-0.60-0.82	-0.048	-0.448-0.381	0.894	Paired Wilcoxon signed-rank test
GFI	Gemini vs Doubao	30	0.34	-0.42-1.05	0.65	-0.45-1.05	0.196	-0.209-0.587	0.709	Paired Wilcoxon signed-rank test
GFI	Copilot vs DeepSeek	30	-0.48	-1.41-0.49	0.00	-1.20-0.30	-0.227	-0.624-0.201	0.709	Paired Wilcoxon signed-rank test
GFI	Copilot vs Doubao	30	0.04	-0.85-0.86	0.10	-0.40-1.16	0.110	-0.294-0.522	0.802	Paired Wilcoxon signed-rank test
GFI	DeepSeek vs Doubao	30	0.52	-0.19-1.19	0.61	-0.60-1.40	0.348	-0.058-0.708	0.709	Paired Wilcoxon signed-rank test
SMOG	ChatGPT vs Gemini	30	-0.99	-1.84-0.01	-1.35	-1.77--0.74	-0.622	-0.961--0.234	0.008	Paired Wilcoxon signed-rank test
SMOG	ChatGPT vs Copilot	30	-0.59	-1.56-0.37	-0.66	-1.51--0.16	-0.368	-0.742-0.024	0.134	Paired Wilcoxon signed-rank

Metric	Comparison	N pairs	Mean difference	Mean difference 95% CI	Median difference	Median difference 95% CI	Rank-biserial correlation	Rank-biserial 95% CI	Adjusted *P*	Note
										test
SMOG	ChatGPT vs DeepSeek	30	-1.04	-1.97--0.05	-1.38	-2.13--0.46	-0.523	-0.850--0.140	0.026	Paired Wilcoxon signed-rank test
SMOG	ChatGPT vs Doubao	30	0.56	-0.47-1.58	0.36	-0.21-1.14	0.315	-0.071-0.697	0.202	Paired Wilcoxon signed-rank test
SMOG	Gemini vs Copilot	30	0.40	-0.56-1.28	0.34	-0.38-0.80	0.228	-0.200-0.602	0.322	Paired Wilcoxon signed-rank test
SMOG	Gemini vs DeepSeek	30	-0.05	-0.89-0.76	-0.04	-0.59-0.47	-0.043	-0.454-0.359	0.845	Paired Wilcoxon signed-rank test
SMOG	Gemini vs Doubao	30	1.55	0.53-2.57	1.72	0.90-2.32	0.654	0.290-0.979	0.006	Paired Wilcoxon signed-rank test
SMOG	Copilot vs DeepSeek	30	-0.45	-1.16-0.23	0.06	-1.21-0.36	-0.224	-0.600-0.222	0.322	Paired Wilcoxon signed-rank test
SMOG	Copilot vs Doubao	30	1.15	0.67-1.61	1.00	0.64-1.80	0.802	0.523-0.987	<0.001	Paired Wilcoxon signed-rank test
SMOG	DeepSeek vs Doubao	30	1.60	0.97-2.25	1.64	0.49-2.08	0.841	0.587-0.987	<0.001	Paired Wilcoxon signed-rank test

Metric	Comparison	N pairs	Mean difference	Mean difference 95% CI	Median difference	Median difference 95% CI	Rank-biserial correlation	Rank-biserial 95% CI	Adjusted *P*	Note
FRES	ChatGPT vs Gemini	30	7.14	3.54-11.35	5.00	0.98-9.00	0.717	0.397-0.936	0.012	Paired Wilcoxon signed-rank test
FRES	ChatGPT vs Copilot	30	4.37	0.79-7.75	5.50	1.42-9.50	0.482	0.097-0.806	0.054	Paired Wilcoxon signed-rank test
FRES	ChatGPT vs DeepSeek	30	5.00	-0.19-9.75	6.00	1.25-11.00	0.508	0.118-0.852	0.054	Paired Wilcoxon signed-rank test
FRES	ChatGPT vs Doubao	30	0.79	-4.31-6.24	1.50	-3.00-5.00	0.064	-0.355-0.473	0.785	Paired Wilcoxon signed-rank test
FRES	Gemini vs Copilot	30	-2.77	-6.39-0.63	-1.00	-5.00-4.00	-0.195	-0.583-0.256	0.519	Paired Wilcoxon signed-rank test
FRES	Gemini vs DeepSeek	30	-2.15	-7.62-2.92	0.00	-4.00-4.50	-0.062	-0.467-0.350	0.785	Paired Wilcoxon signed-rank test
FRES	Gemini vs Doubao	30	-6.36	-10.84--2.30	-3.50	-7.46-0.00	-0.495	-0.796--0.116	0.054	Paired Wilcoxon signed-rank test
FRES	Copilot vs DeepSeek	30	0.62	-4.51-5.33	1.00	-3.00-7.68	0.166	-0.249-0.548	0.543	Paired Wilcoxon signed-rank test

Metric	Comparison	N pairs	Mean difference	Mean difference 95% CI	Median difference	Median difference 95% CI	Rank-biserial correlation	Rank-biserial 95% CI	Adjusted *P*	Note
FRES	Copilot vs Doubao	30	-3.58	-8.38-1.39	-3.50	-7.00--0.50	-0.363	-0.723-0.026	0.140	Paired Wilcoxon signed-rank test
FRES	DeepSeek vs Doubao	30	-4.21	-9.14-1.18	-6.50	-10.06--1.00	-0.470	-0.828--0.085	0.061	Paired Wilcoxon signed-rank test

Note: Prespecified exploratory paired Wilcoxon signed-rank comparisons were conducted regardless of omnibus significance, with Benjamini-Hochberg correction within each outcome. Paired mean/median differences and rank-biserial correlations are reported with bootstrap 95% CIs. Existing numerical values are retained unchanged.
